# Testing Cessation Messages for Cigarette Package Inserts: Findings from a Best/Worst Discrete Choice Experiment

**DOI:** 10.3390/ijerph15020282

**Published:** 2018-02-06

**Authors:** James F. Thrasher, Farahnaz Islam, Rachel E. Davis, Lucy Popova, Victoria Lambert, Yoo Jin Cho, Ramzi G. Salloum, Jordan Louviere, David Hammond

**Affiliations:** 1 Department of Health Promotion, Education & Behavior, Arnold School of Public Health, University of South Carolina, Columbia, SC 29208, USA; RDAVIS@mailbox.sc.edu (R.E.D.); vlambert@email.sc.edu (V.L.); ycho@email.sc.edu (Y.J.C.); 2 Department of Biostatistics and Epidemiology, Arnold School of Public Health, University of South Carolina, Columbia, SC 29208, USA; fislam@email.sc.edu; 3 Division of Health Promotion and Behavior, School of Public Health, Georgia State University, Atlanta, GA 30302, USA; lpopova1@gsu.edu; 4 Department of Health Outcomes & Policy, College of Medicine, University of Florida, Gainesville, FL 32610, USA; rsalloum@ufl.edu; 5 Institute for Choice and School of Marketing, University of South Australia, Adelaide, SA 5001, Australia; louviere.jordan@gmail.com; 6 School of Public Health & Health Systems, University of Waterloo, Waterloo, ON N2L3G1, Canada; david.hammond@uwaterloo.ca

**Keywords:** tobacco control, health communication, smoking cessation, health policy

## Abstract

This study assessed smokers’ responses to different smoking cessation topics and imagery for cigarette package inserts. Adult smokers from Canada (*n* = 1000) participated in three discrete choice experiments (DCEs): DCE 1 assessed five cessation benefit topics and five imagery types; DCE 2 assessed five messages with tips to improve cessation success and five imagery types; DCE 3 assessed four reproductive health benefits of cessation topics and four imagery types. In each DCE, participants evaluated four or five sets of four inserts, selecting the most and least motivating (DCEs 1 & 3) or helpful (DCE 2) for quitting. Linear mixed models regressed choices on insert and smoker characteristics. For DCE 1, the most motivating messages involved novel disease topics and imagery of younger women. For DCE 2, topics of social support, stress reduction and nicotine replacement therapy were selected as most helpful, with no differences by imagery type. For DCE 3, imagery influenced choices more than topic, with imagery of a family or a mom and baby selected as most motivating. Statistically significant interactions for all three experiments indicated that the influence of imagery type on choices depended on the message topic. Messages to promote smoking cessation through cigarette pack inserts should consider specific combinations of message topic and imagery.

## 1. Introduction

In 2000 Canada was the first country to mandate pictorial health warnings on cigarette packs; since then, over 100 countries have implemented them [1]. Pictorial warnings generally illustrate the consequences of tobacco use in order to prevent tobacco use in nonsmokers and to motivate smokers to quit. Evidence from across the globe to date indicates that prominent, pictorial warnings with fear arousing content effectively promote smoking cessation intentions and behaviors [2,3]. However, the Extended Parallel Process Model (EPPM) asserts that the impact of these fear-arousing messages could be further enhanced by including complementary messages that enhance response efficacy and self-efficacy [4,5]. Response efficacy is comprised of beliefs that some actions (such as quitting smoking) might avert the threat of diseases caused by smoking. Messages communicating response efficacy typically focus on benefits of quitting smoking. Self-efficacy comprises beliefs that one is capable of carrying out these responses (i.e., one can quit smoking). Messages aimed at raising self-efficacy frequently communicate tips about how to quit smoking.

A lesser known characteristic of the Canadian warning label policy is its requirement for “inserts”, which are small, printed leaflets inside of cigarette packs that contain messages about the benefits of quitting (e.g., response efficacy) and behavioral recommendations to increase successful smoking cessation (e.g., self-efficacy) (see: www.tobaccolabels.ca/countries/canada). Observational studies of Canadian smokers indicate that those who read inserts are more likely to have stronger downstream self-efficacy to quit and are more likely to subsequently make a quit attempt, including attempts that last for at least a month [6,7]. Prior research on pictorial warnings about smoking-related harms has identified some types of imagery as more effective than other types, such as graphic illustrations of bodily harm (e.g., open heart surgery) compared to symbolic representations of risk (e.g., a bomb to represent pending heart attack) [8,9,10,11]. To our knowledge, however, no research has assessed the effectiveness of pictorial complements for efficacy-enhancing messages, whether for promoting response efficacy or self-efficacy to quit.

Given the theoretical and empirical support for the Canadian labeling policy of complementing pictorial warnings with inserts containing efficacy messages, there is a need for research on how to best capitalize on the expanded space for public health messages that inserts provide. The current experimental study assessed smokers’ responses to different imagery and efficacy message topics that could be used for inserts that aim to promote smoking cessation, including which smoker characteristics influenced the perceived effectiveness of messages.

### 1.1. Topics for Efficacy Messages about Smoking Cessation

Smokers’ responses to different types of messages that target efficacy beliefs around smoking cessation have rarely been studied. Observational research among Canadian smokers has assessed attention to and effects of inserts [6,7,12]; however, these studies did not distinguish the effects of different insert content, which as of 2012 included four inserts with messages about cessation benefits and six with quitting tips. An experimental study with US smokers on insert content [13] found that smokers perceived cessation benefit messages as more effective than quitting tips messages. However, no study of which we are aware has systematically assessed the effectiveness of specific topics within these two types of message categories.

### 1.2. Pictorial Imagery and Smoking Cessation Messages

Pictorial imagery is a potentially important characteristic of messages, as it can enhance message recall [14,15] and persuasiveness [16]. Studies have consistently demonstrated that cigarette package warnings with images are more effective than text-only warnings in promoting knowledge of tobacco-related risks and encourage smoking cessation, whether in experimental studies [3], observational studies [2], or randomized behavioral trials [17,18,19]. These studies have primarily assessed imagery that illustrates smoking-related harms, where negative emotional responses can play an important role in mediating the effects of warnings on smoking cessation behaviors [18,20,21]. For warnings that emphasize the negative consequences of smoking (i.e., “loss frame” messages), imagery that graphically illustrates the harms of smoking or that shows personal suffering from these harms is consistently rated by smokers and youth as more effective than imagery that symbolically represents risk (e.g., a bomb to represent a pending heart attack) [8,9,10,11,22]. Such studies have provided the foundation for WHO policy recommendations about warning label content [23].

Research on smokers’ responses to imagery with efficacy messages should inform recommendations for the most effective inserts to promote smoking cessation. Consistent with research on cigarette warnings, experimental research finds that smokers perceive both cessation benefit messages and cessation tips as more effective when they include pictorial imagery than when they do not [13]. However, to our knowledge, no published research has systematically assessed specific types of imagery to accompany such messages. Compared to loss-frame warnings about smoking-related harms, these messages are more positive: cessation benefit messages focus on positive outcomes associated with cessation (i.e., “gain frame”), and quitting tips promote specific cessation strategies to enhance smokers’ self-efficacy to quit. Some pictorial warning studies have found that smokers and youth rate messages about cessation as less effective than messages about smoking-related risks [9,10]. This may be because the type of imagery that “fits” cessation messages involves less negative emotional arousal. Because efficacy messages are less likely to work through the channel of negative affect, research should determine which types of pictorial imagery will best enhance efficacy message effects.

### 1.3. Concordance and Message Effects

Message effects can depend on the extent to which message receiver characteristics are concordant with message attributes. Indeed, messages are believed to be more effective when recipients perceive them as personally relevant [24,25]. Enhancing message relevance is critical to targeted and tailored communication approaches, which frequently match visible characteristics of actors displayed in graphics (e.g., race, sex) with message recipient characteristics [26]. Compared with non-tailored print materials, tailored materials are generally better remembered, read, perceived as relevant and/or credible, and more effective in promoting behavior change [27]. Hence, the visible characteristics of people portrayed in insert messages may influence smokers’ responses to those messages.

Concordance between a message and its recipient may also be due to the textual content of the message. For example, some research has found that cigarette warnings about pregnancy-related harms from smoking are rated as more effective by women of reproductive age than by other groups [28]. Efficacy messages about cessation benefits and quitting tips may be more relevant and effective for smokers who intend to quit or have recently tried to quit, as found for pictorial warnings that generally use loss-framed messages [29,30,31,32]. Since public health messages that use cigarette packs as communication vehicles reach all smokers, it is important to determine which message characteristics are generally effective across all types of smokers, as well as within particular segments, such as those intending to quit.

### 1.4. Objective

The current study used a series of discrete choice experiments (DCEs) to assess Canadian smokers’ preferences for different message content and imagery (adapted from those considered by Health Canada for implementation in 2020) that could be used on newly adopted inserts. Three separate DCEs were conducted in order to assess: (1) cessation benefit messages that targeted a general audience of adult smokers (DCE 1); (2) quitting tips messages that targeted a general audience of adult smokers, but particularly those interested in quitting (DCE 2); and (3) messages about pregnancy-related cessation benefits that targeted smokers of reproductive age (DCE 3). We also assessed the test-retest reliability of DCE 1 and DCE 2 in a subsample of participants who were followed up four weeks later.

## 2. Materials and Methods

### 2.1. Sample

Adult smokers from Canada were recruited through GMI-Lightspeed’s online consumer panel. Invitation emails with a link to the online survey were sent to panel members, who were given a brief study description before providing consent. Eligible participants were 18 to 64 years old, had smoked at least 100 cigarettes in their lifetime, and had smoked cigarettes at least once in the prior 30 days. The first wave of the experiment was conducted between 22 and 31 August 2017. Sample quotas were established to recruit a minimum of 1000 participants, 50% of whom intended to quit smoking within the next 6 months. Four weeks after first participation in the experiments (20–30 September 2017), we recontacted participants to re-administer the experiments. GMI-Lightspeed has several quality control measures in place to ensure participants are engaged and provide thoughtful responses, including removal of panelists who complete the questionnaire too quickly (i.e., within 2/5ths of the median time) and regular screening of panelists with short batteries of questions and associated algorithms to identify and eliminate from the panel those who do not provide truthful, engaged responses. Participants were provided compensation that is standard for GMI-Lightspeed (i.e., baseline range = $0.30–$0.65; follow-up range = $1.00–$3.00). The study protocol received ethics approval from the IRB at the University of South Carolina (Pro00054788).

### 2.2. Experimental Protocol

We used discrete choice experiments (DCEs), which have been used extensively in transportation studies, environmental economics, and marketing [33,34,35]. DCE protocols use full factorial or fractional factorial designs to create sets of alternatives from which participants choose. Their key strength is the ability to simultaneously assess the effects of specific stimulus characteristics on decision-making independent of other characteristics that are manipulated, while also providing an indication of the relative impact of each characteristic on choices [36]. The tobacco industry uses DCEs in premarket research [37,38,39], and, in international litigation, tobacco industry experts highlight how DCEs are less biased (e.g., reduced demand effects) than the other methods that public health researchers use to study tobacco packaging and labeling [40]. A growing number of tobacco research studies have used DCE methods to assess the effects of different characteristics of cigarette pack design elements, cigarette branding, cigarette sticks, and health warnings [41,42,43,44]. However, none of these studies has assessed the reliability of DCE methods. Assessing the test-retest reliability of DCEs in the context of pre-market testing of inserts may be particularly important given that smokers are repeatedly exposed to inserts and the effectiveness of some message characteristics may change over time. Our approach involved “best-worst” scaling, which asks participants to choose stimulus configurations they prefer *most* and *least*, thereby increasing the precision of estimates and statistical power for assessing the reliability of DCE data [45].

**Figure 1:**
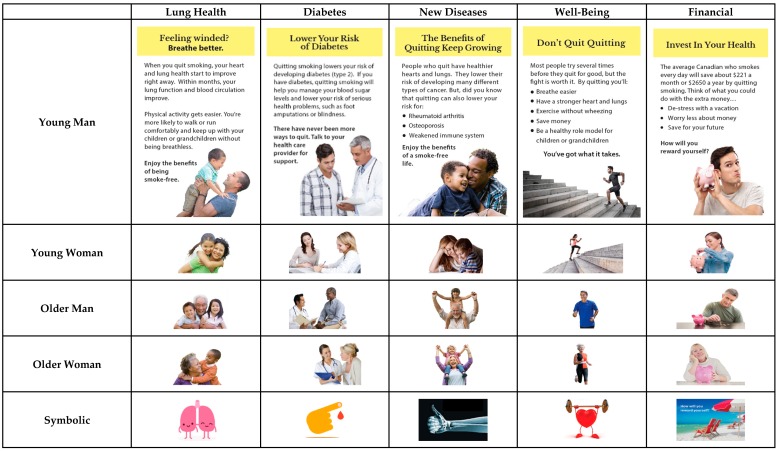
Topic and imagery for cessation benefit messages in DCE 1.

**Figure 2:**
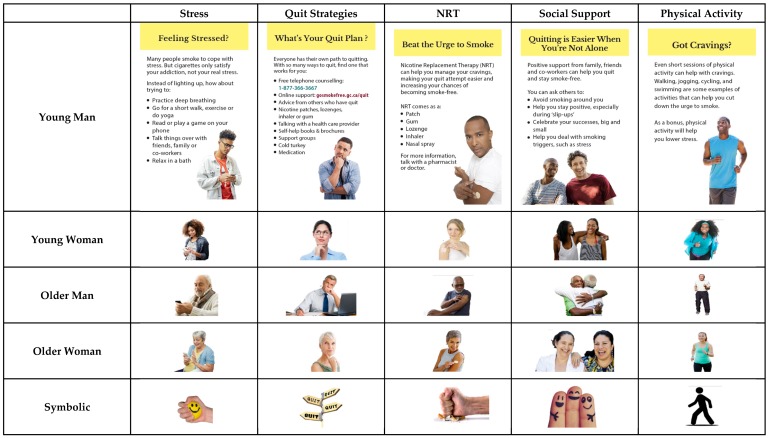
Topic and imagery for quitting tips messages in DCE 2.

**Figure 3:**
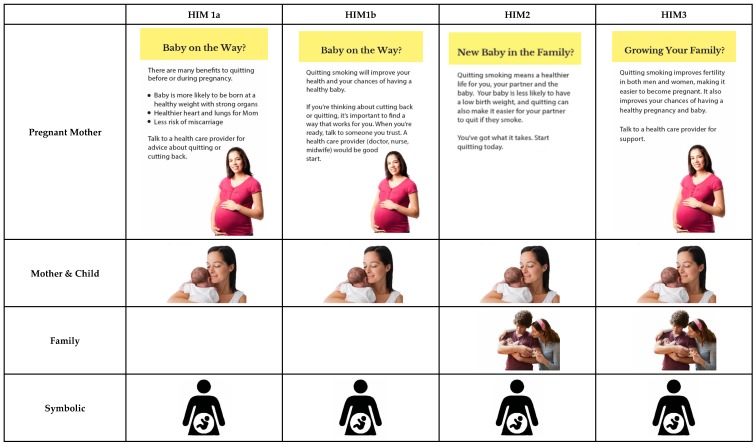
Topics and imagery for reproductive health benefit messages in DCE 3.

Participants evaluated three blocks of material that corresponded to three DCEs. In each DCE, participants evaluated four or five sets of four inserts (i.e., “choice sets”), selecting the most and least motivating (DCEs 1 & 3) or helpful (DCE 2) for quitting. DCE 1 on cessation benefit messages unrelated to reproductive health involved a 5 × 5 within-subjects design (with a between-subjects element due to random assignment to different blocks of choice sets): five distinct topics (i.e., avoiding diabetes; avoiding arthritis, osteoporosis, and weakened immune system [new disease]; improving lung health; enhancing wellbeing; financial benefits of quitting) and five types of accompanying imagery (i.e., older male, younger male, younger female, older female, and non-human symbolic representation) were tested (See Figure 1). DCE 2 on quitting tips messages also involved a 5 × 5 within-subjects design (and between-subjects from random assignment to blocks): five messages about strategies to quit (i.e., stress reduction, physical activity, social support, nicotine replacement therapy, list of cessation strategies) and the same five imagery types as in DCE 1 were assessed (See Figure 2). In DCE 3 there were four different messages that addressed benefits of quitting before or during pregnancy (i.e., health of mom and baby; health of mom and baby, with cessation tips; health of mom, dad and baby; fertility of mom and dad, as well as healthy pregnancy and baby) and four image types (i.e., pregnant mom, mom with baby, mom and dad with baby, symbolic figure of pregnant mom) (see Figure 3). The use of multiple messages on different topics aimed to increase the generalizability of our findings beyond single messages typically used in experimental studies, which is recommended for media effects research [46].

Insert message topics were selected and adapted from those developed by Health Canada as part of the process of selecting message content for inserts that will be implemented in 2020. Health Canada and the research team worked with a graphic designer to identify a range of possible images that fit each message and the typologies used (e.g., males and females that appeared younger or older than 40 for DCEs 1 & 2; symbolic images for messages in all DCEs).

**Figure 4:**
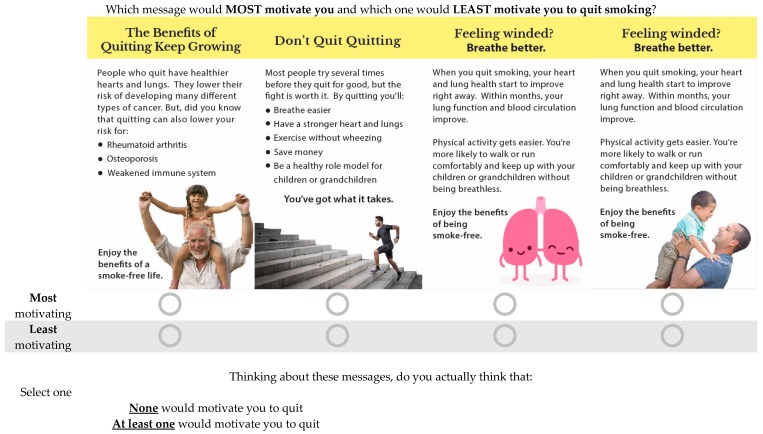
Example choice set for DCE 1.

For all participants, DCE 1 was followed by DCE 2 and DCE 3. For both DCE 1 and DCE 2, all 25 message combinations were used. Fifty different “choice sets” were used, each with four contrasting inserts, such that the alternatives were pairwise independent of each other across choice sets (see Figure 4 for example choice set). To reduce response burden in both DCE 1 and 2, participants were randomized to evaluate one of ten blocks, each of which included five choice sets. For DCE 3, all meaningful combinations of topics and images were used. Whereas DCEs 1 and 2 used different images for each topic, DCE 3 used the same images across topics. However, there was only one message for which all four images matched the topic. We could meaningfully match the other three messages with three of the four possible images. This resulted in 13 distinct topic and image combinations, and 13 different choice sets with four contrasting inserts. The alternatives were pairwise independent of each other across choice sets. Participants were randomized to evaluate one of three blocks that contained either 4 or 5 choice sets. Hence, at baseline, each participant evaluated 14 or 15 choice sets (i.e., 5 for DCE 1; 5 for DCE 2; and 4 or 5 from DCE 3), with the choice sets presented in random order within each experiment. The final insert configurations, choice sets, and blocks of choice sets can be found in Supplementary Tables S1–S3.

Participants who were successfully re-contacted (58%, *n* = 582) were assigned to the same blocks of material that they evaluated at baseline in DCEs 1 and 2. Responses to DCE 3 stimuli were not assessed due to concerns about survey length and because of expectations regarding the substantially smaller analytic sample both due to attrition and exclusion after considering participants who found no messages in DCE to be motivating (because of the narrow focus on reproductive health topics).

### 2.3. Measures

Before beginning the experimental protocol, all participants were told that they would be shown health information that could appear inside cigarette packages, either on small paper leaflets or printed on the inside of packages. Both ways of delivering insert information are used in Canada, depending on whether the package is a “flip top” or “slider pack” (i.e., pack opens like a drawer), respectively.

#### 2.3.1. Dependent Variables

For each choice set, participants were presented with four inserts, and for DCEs 1 and 3 they were asked “*Which insert would MOST motivate you and LEAST motivate you to quit smoking?*”, with participants choosing one insert as “most motivating” and one as “least motivating”, with mutually exclusive options allowed for each choice set (see Figure 4 and Supplementary Figures S1 and S2). Afterwards, participants were asked “*Do you actually think that: (a) None would be motivating if you decided to quit, or (b) At least one would be motivating if you decided to quit?*” For DCE 2, participants were asked “*Which insert would be MOST helpful and which would be LEAST helpful for you if you decided to quit smoking?*” with mutually exclusive choices allowed for each insert. Then, participants were asked “*Thinking about these inserts, do you actually think that: (a) None would be helpful if you decided to quit, or (b) At least one would be helpful if you decided to quit?*” Participants could view each choice set for as long as they wished. For each choice set, the insert selected as most helpful/motivating to quit smoking was assigned a value of 1, and the least helpful/motivating to quit was assigned a value of −1. The remaining inserts in that set were assigned a value of 0. If the participant indicated that none would be helpful/motivating, all inserts in that choice set were assigned a value of 0.

#### 2.3.2. Independent Variables

Insert characteristics (i.e., message topics, imagery types) were effects coded such that coefficients reflected deviations of the group from the grand mean. Participant sociodemographics included age group in years (18–29, 30–39, 40–49, 50–64), sex (male, female), education (high school or less, college or some university, completed university or higher), and income (under $30,000, $30,000–59,999, $60,000–99,999, $100,000 and over). Smoking-related variables included: smoking frequency (every day; some days); nicotine dependence as determined by the heaviness of smoking index (HSI, a function of average cigarettes per day and time to first cigarette after waking [47]; intention to quit, with responses dichotomized into quit intention in the next 6 months or not [48]; and at least one quit attempt in the prior four months (yes, no).

### 2.4. Data Analysis

Within each DCE, participants who indicated that none of the inserts would be helpful/motivating for all choice sets they evaluated were excluded from the analysis for that DCE. This exclusion was due to their not contributing any meaningful information for assessing specific insert characteristics that influence choice. Using chi-square tests, we compared the demographic and smoking-related characteristics of participants who found no insert message to be motivating/useful (excluded) with those who found at least one insert message to be motivating/useful (analytic sample). Omnibus chi-square tests were used to assess whether participant characteristics differed across the blocks of stimuli to which they were randomized.

We used mixed linear regression to control for repeated measures when analyzing each DCE’s analytic sample [45]. Dependent variables reflected the choice of an insert as motivating or helpful to quit, depending on the DCE. Independent variables included insert characteristics (topic, imagery type), controlling for block assignment, sociodemographics, and smoking-related participant characteristics. The relative impact of each insert characteristic on choice was calculated using a utility range (i.e., the difference between each characteristic’s highest and lowest estimated part-worth utility or estimated effect on choices), divided by the sum of all the characteristics’ utility ranges for a given outcome. We also tested for concordance effects by assessing interactions between message imagery type and participant characteristics (i.e., sex, age). Age groups were dichotomized (18–39; 40–64) because the young vs. old contrast in the stimuli were based whether the person portrayed in the image clearly appeared younger or older than 40. Because being younger than 40 generally reflects female reproductive age, this contrast was also meaningful for DCE 3. Finally, we assessed interactions between message topic and imagery type.

We conducted these analyses for the entire baseline sample, as well as for the subsample that was followed up and repeated DCEs 1 and 2. For the sample that was followed up, the consistency of choices in DCEs 1 and 2 were based on Cohen’s kappa. All data analyses were conducted using Stata v. 13.1 (StataCorp LLC, College Station, TX, USA).

## 3. Results

### 3.1. Sample Characteristics

In the baseline sample (*n* = 1000; see Table 1), most participants were 40 or older (59%), female (58%), and daily smokers (78%). For all three DCEs, no statistically significant differences were found in participant characteristics across blocks to which participants were randomized (results not shown). The analytic samples differed for each DCE (see explanations in Section 3.2, Section 3.4 and Section 3.6).

### 3.2. DCE 1—Cessation Benefit Messages for a General Audience

The proportion of participants who indicated that at least one of the DCE 1 messages motivated them to quit was 80% (*n* = 804). Those who opted out of all choice sets they evaluated were less likely to intend to quit (37% vs. 54%; *p* < 0.001) or to have tried to quit recently (32% vs. 49%; *p* < 0.001) compared to those who selected at least one insert.

### 3.3. Effects of Specific Cessation Benefit Message Topics and Imagery on Choice

**Figure 5:**
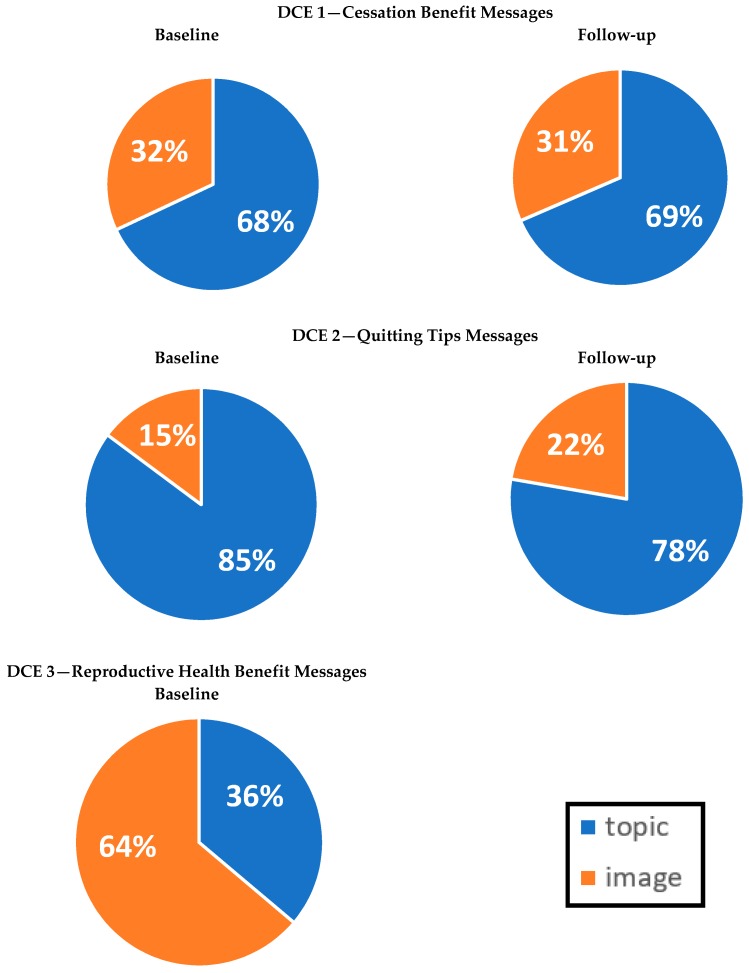
Relative importance * of attributes on choice of insert (* Relative importance was calculated using a utility range (i.e., the difference between each characteristic’s highest and lowest estimated part-worth utility or estimated effect on choices), divided by the sum of all the characteristics’ utility ranges for a given outcome. DCE 3 was conducted only at baseline).

The relative importance of message topic on choice (see Analysis section) was much higher than for imagery (68% vs. 32%; see Figure 5). Correspondingly, the overall effects of message topics on message selection were statistically significant (*p* < 0.001), whereas the overall effects of imagery were not (*p* = 0.15; see Table 2). Message topics of diabetes (*b* = 0.014) and new diseases (*b* = 0.015) were significantly more likely to be selected as motivating than the average of all messages, whereas topics on wellbeing (*b* = −0.013) and financial benefits from cessation (*b* = −0.016) were identified as less motivating. No statistically significant influences for imagery type were found, although imagery of younger women was only marginally non-significant (*b* = 0.008, *p* = 0.076).

Interactions between participants’ sex and imagery was marginally non-significant (*p* = 0.052). Males selected messages with imagery of older women to be less motivating (*b* = −0.015) and symbolic imagery to be more motivating (*b* = 0.016). No imagery was selected as superior for females.

The overall interaction between participants’ age and imagery type was marginally non-significant (*p* = 0.056). However, when examining specific coefficients, older participants selected messages with imagery of younger women to be more motivating (*b* = 0.013) and imagery of older men to be less motivating (*b* = −0.014). The overall interaction between topic and imagery was statistically significant (*p* < 0.001), indicating that the effectiveness of any particular image type depended on the topic (see Supplementary Figure S3).

### 3.4. DCE 2—Quitting Tips Messages for a General Audience

More than three-quarters (78%; *n* = 778) of the sample found at least one message helpful. Compared to those who chose at least one insert as helpful, those who found no messages helpful were more likely to have lower education (i.e., 21% vs. 34% with university degree or more; *p* < 0.001), be more nicotine dependent (HSI: 3.28 vs. 3.01, *p* = 0.008), be less likely to intend to quit (35% vs. 55%; *p* < 0.001) or to have tried to quit recently (32% vs. 49%; *p* < 0.001).

### 3.5. Effects of Specific Quitting Tips Message Topics and Imagery on Choice

The relative impact of message topic on choice was substantially higher than for image type (85% vs. 15%; see Figure 5). Message topic effects on selection of messages were statistically significant (*p* < 0.001; see Table 3). The topic of social support was selected as significantly more helpful (*b* = 0.017) and on average the topic that listed cessation strategies was selected as less helpful (*b* = −0.046) than other messages. Imagery type did not have a significant effect on choice, whether assessed overall or individually.

Interactions between imagery type and participant sex and age were not significant (*p* = 0.584 and *p* = 0.447, respectively), although on average older participants selected messages with imagery of the young man to be less helpful (*b* = −0.014). The overall interaction between message topic and imagery was statistically significant (*p* < 0.001), indicating that the effectiveness of imagery was contingent on message topic (see Supplementary Figure S4).

### 3.6. DCE 3—Messages on Reproductive Health Benefits of Cessation

In DCE 3, about half of the baseline sample (57%; *n* = 577) found at least one message motivating to quit. Compared to those who found at least one message motivating, those who did not were more likely to be older (i.e., 30% vs. 51% 18 to 39 years old, *p* < 0.001), have lower education (i.e., 25% vs. 35% with university or more, *p* < 0.001), have lower income (i.e., 29% vs. 20% with household income <$30,000/year, *p* = 0.005), smoke more often (82% vs. 73% daily smokers, *p* = 0.001), have greater nicotine dependence (HSI = 3.24 vs. 2.95, *p* < 0.001), have tried to quit recently (52% vs. 36%, *p* < 0.001), and be less likely to intend to quit (40% vs. 58%, *p* < 0.001).

### 3.7. Effects of Specific Message Topics and Imagery on Choice

Imagery type had a larger influence on message helpfulness than message topic (62% vs. 38%; see Figure 5); nevertheless, both message topic and imagery type had statistically significant overall effects on choices (*p* < 0.001 for both; see Table 4).

The message topic of healthy mom and baby (*b* = 0.093) and of mom, dad and baby (*b* = 0.031) were selected as significantly more motivating than the average, whereas the topic of fertility (*b* = −0.118) was significantly less motivating (see Table 1). Messages with imagery of real people, whether of a mom and baby (*b* = 0.107), or a mom, dad and baby (*b* = 0.122) were selected as significantly more motivating than the average, whereas the symbolic figure of a pregnant woman (*b* = −0.252) were selected as significantly less motivating (see Table 4).

The overall interaction between participant sex and message imagery was statistically significant (*p* < 0.001). However, the pattern of responses was similar for males and females (see Table 1), except that males had particularly strong responses to messages with imagery that included a dad (*b* = 0.223 and 0.045, respectively), and females selected messages with imagery of a pregnant woman (*b* = 0.045) while males did not.

Interactions between age and imagery were found to be significant (*p* < 0.001). However, the primary difference between younger and older groups concerned the somewhat stronger effects of imagery on message selection amongst older compared to younger participants. No differences in direction of effect or statistical significance were found (see Table 1). Finally, the impact of imagery on message selection depended on message topic, as evinced by the statistically significant interaction between topic and imagery (*p* < 0.001; see Supplementary Figure S5).

### 3.8. Reliability of DCE

Four weeks after the baseline experiments, 58% of participants were successfully re-contacted (*n* = 582) and re-evaluated the same blocks of inserts they evaluated in DCEs 1 and 2 at baseline. Compared to the baseline sample, those who were followed up were more likely to be older than 40 (67% vs. 59%; *p* = 0.002) and less likely to intend to quit (43% vs. 50%; *p* = 0.001). Results were similar with regard to differences between the analytic and excluded samples due to opting out (results not shown). For both DCEs 1 and 2, the percent agreement in choice was 94%, with a Kappa statistic of 0.38 for DCE 1 and 0.37 for DCE 2, which indicates a moderate level of reliability.

## 4. Discussion

This study assessed cessation benefit and quitting tips message topics and imagery most likely to be effective for the innovative Canadian policy of using cigarette package inserts complement pictorial warnings with graphic illustrations of smoking-related harms. We found that the vast majority of smokers identified at least one general cessation benefit message to be motivating (80%) or a quitting tip message to be helpful (78%). As expected, these smokers were more likely to intend to quit or have recently tried to quit when compared to those who found no message motivating or helpful. It is noteworthy, however, most smokers who did not intend to quit also found these messages to be effective (DCE 1 = 71%; DCE 2 = 66%). While theories of health behavior, such as the theory of planned behavior [49,50], highlight the role of intention as the gateway to behavior change, observational studies have found that the association between insert exposure and subsequent cessation behavior appears at least partly independent of quit intentions [6,7]; hence, attention to insert information may become useful at the moment when one finally decides to quit—which may change in unpredictable ways over time [51].

Finding cessation benefit or quitting tips messages to be helpful was inversely associated with SES indicators (income and education, respectively). This may reflect SES-related barriers (e.g., lower support, lower access, higher stress) that can cause communication campaigns to exacerbate health inequalities [52]. To better determine the health equity impact of these kinds of messages, it will be important to assess their effectiveness in the context of fear arousing warning labels, as studies have found minimal to stronger responses to fear-appeals among relatively lower SES smokers [8,22,53,54,55,56,57,58].

Smokers’ evaluations of messages about reproductive health benefits of cessation exhibited a similar pattern: smokers with cessation intentions, recent quit attempts and higher SES were more likely to select at least one message as motivating. The percentage of smokers in this group was lower than for the aforementioned messages that targeted a broader audience, yet more than half of smokers still found at least one of these reproductive health messages to be effective (58%). As expected, younger smokers were more likely to be in this group than to be unaffected, likely because of message relevance for people of reproductive age; however, females and males were equally likely to be affected, inconsistent with results for loss-frame messages on reproductive health that showed stronger responses among females [28]. This may be because men were mentioned in at least some, although not all, of the messages evaluated, therefore providing them with relevant content that is often not present in warning labels.

When assessing cessation benefit and quitting tips messages with topics that targeted the general audience of smokers (i.e., DCE 1 and DCE 2), message topics explained more variation in choices than imagery types, whereas the opposite was found for reproductive health messages (DCE 3). The contrasting results between the first two DCEs and the third is likely due, in part, to the broader range of topics used for the more general cessation messages relative to the narrower range of reproductive health topics. Furthermore, different images were used within any particular image type category (e.g., young men) across general efficacy topics for the first two DCEs to make the imagery “fit” general efficacy message topics—this contrasts with the fixed images within each imagery type category across the reproductive health topics (see Figure 1, Figure 2 and Figure 3). Hence, while any particular image of a young woman may work better or worse than another image within that category in DCE 1 and DCE 2, tighter control and assessment of the particular image in DCE 3 likely contributed to the stronger effect of imagery on choice. Indeed, we found statistically significant interactions between topic and image, indicating that the most effective image type depended on the topic (see Supplementary Figures S3–S5). For DCE 2, no image type worked better than the others and only one, relatively weak general tendency of a particular imagery type (i.e., young woman) worked better than others for DCE 1. Significant interactions indicate a better fit between image types for some topics than for others, consistent with other research suggesting that the congruence between topic and imagery in pictorial warnings influences attention and recall [59].

We found mixed support for the importance of image congruence with smokers’ sex and age. For general cessation messages (DCEs 1 & 2), females were relatively uninfluenced by any particular imagery type whereas males had weaker responses to messages that portrayed an older woman. For reproductive health benefit messages (DCE 3), image congruence mattered more, with females having stronger responses to pregnancy imagery than males and males having stronger responses than females to family imagery that included a father. Smoker age mattered when considering responses to general cessation benefit messages (DCE 1; older smokers found younger women more motivating and older men less motivating), but not for quitting tips messages (DCE 2). For the reproductive health benefit messages, no striking differences were found by age, except that imagery appeared to have a somewhat stronger effect amongst older than younger participants. Participants across all age groups agreed that the symbolic figure of the pregnant woman was less motivating than the imagery of real people. Contrary to expectations, however, symbolic imagery was quite effective for some general efficacy topics (see Supplementary Figures S3–S5), which contrasts with their weak effects found in more typical, “loss-framed” pictorial warning messages [8,9,10,11,22]. Hence, symbolic representations of cessation messages may be relatively more effective, perhaps due to the more abstract nature of cessation topics and the relative difficulty of pairing these topics with personified imagery. In the end, we found relatively weak evidence for any particular type of imagery as being most effective, whether assessed overall or within specific age or sex strata of smokers. However, this conclusion should be evaluated alongside our results indicating that type of image that is most effective appears to depend on the message topic.

Our study generally supported the reliability of DCE results over time. The factors associated with indicating at least one insert was motivating/helpful and the variance explained by each message attribute were consistent across the baseline and follow-up DCEs (Figure 2). Although the Kappa statistic indicated only a fair level of agreement (*K* = 0.36–37), such scores are not uncommon given the paradoxical nature of the Kappa statistic [60]; indeed, the observed percent of agreement across DCEs was high (94%), indicating that choices are relatively stable over time. Qualitative comparison of the statistical significance and the direction of coefficients also found that most coefficients (49/60 = 82%) led to the same conclusions across the baseline and follow-up assessments. Some discrepancies (*n* = 2) appeared due to reduced statistical power in the follow-up study due to attrition. Remaining discrepancies (*n* = 9) also may be due to statistical power issues (i.e., topics that elicited weaker responses became non-significant in the follow-up DCE 2, whereas the stronger topics did not), as well as random fluctuation in responses perhaps due to the hypothetical nature of the task performed. Another explanation, however, concerns repeated exposure. Learning of insert content may make inserts less effective over time—for example, the topic of new diseases became statistically non-significant at follow-up after being motivating to quit at baseline. Future research should more squarely explore the impact of learning, since naturalistic insert exposure involves repetition. Indeed, some prior research has found that attention to inserts with cessation messages increases over time, even while attention to warnings wears out, suggesting that some insert message characteristics may become more salient over time [7].

This study has a number of limitations, including its reliance on self-reported choice to evaluate message effectiveness. While DCEs may do a better job of obscuring study intent and reducing demand effects than other study designs, our use of a relatively limited set of message attributes (topic and imagery) may have lessened this advantage. Social desirability bias may have led to overestimation of the percentage of smokers who would be influenced by these messages. Furthermore, the images we used to represent each category were not the same for each topic and therefore did not involve tight experimental control—however, doing this would have resulted in highly variable “fit” of imagery across topics. Even so, our results indicated that perceived message effectiveness depended on the specific combination of imagery and topic. Our stimuli were presented via a relatively unrealistic online modality, although some evidence indicates that online experiment results are similar to those found when presenting warnings in person and on mock cigarette packs [8,9,10,11], as well as when assessing responses after implementation [61]. Our study went some way towards assessing naturalistic repeated exposure through its assessment of reliability and potential differences in responses to messages over time—nevertheless, experimental studies that involve repeated exposure under more naturalistic conditions may be necessary to determine real effects of message characteristics. Still, results from randomized clinical trials with pictorial warnings [17,18,19] have generally found results that are consistent with those found in experimental [3] and observational studies [2]. We did not collect information on some participant characteristics, such as the number of failed quit attempts or health complications from smoking, and these may provide a focus for future research on messages that explicitly address those issues. Finally, future research should also consider conducting research with general populations of smokers in order to determine the consistency of effects across key populations, particularly more disadvantaged panels that are underrepresented in online consumer panels. Special consideration should be given to smokers with mental health conditions and multiple addictions, given the higher prevalence of smoking in these populations and evidence that they respond differently to labeling messages than other groups [62].

## 5. Conclusions

In sum, our study found support for the contention that efficacy messages, whether focused on cessation benefits or quitting tips, on cigarette package inserts may help promote smoking cessation, even amongst Canadian smokers who have been exposed to similar messages through inserts for over a decade. Some message topics appeared generally more motivating and helpful than others, although future research in other countries should help determine whether other topics may be more powerful in setting where efficacy messages have not been a critical component of communication campaigns and labeling policies. Furthermore, implementing multiple inserts may allow for targeting of messages to specific groups, particularly vulnerable groups that suffer tobacco-related disparities. In the end, the specific combinations of message topic and imagery worked best across each message domain we assessed, indicating the need for pretesting specific combinations in order to select message characteristics that are most likely to result in maximum effectiveness for smoking cessation.

## Figures and Tables

**Table 1:** Characteristics of study participants.

Characteristics	Baseline Sample	Follow-Up Sample
	(*n* = 1000)	(*n* = 582)
Age		
18–29	16%	11%
30–39	25%	22%
40–49	18%	19%
50–64	41%	48%
Sex		
Male	42%	46%
Female	58%	54%
Education		
High school or less	33%	32%
College or some university	37%	37%
Completed university or higher	31%	32%
Income		
Under $30,000	23%	21%
$30,000–59,999	28%	28%
$60,000–99,999	28%	30%
$100,000 and over	17%	18%
Smoking frequency		
Every day	78%	79%
Some days	23%	21%
HSI (mean ± standard deviation)	3.08 ± 1.36	3.09 ± 1.28
Quit Intention in next 6 months		
Yes	50%	43%
Quit attempt in the past 4 months		
Yes	45%	40%
DCE 1—cessation benefits		
Analytic sample *	80%	78%/72%
DCE 2—quitting tips		
Analytic sample **	78%	77%/73%
DCE 3—reproductive health benefits		
Analytic sample *	58%	53%/NA

* At least one message was motivating to quit; therefore data could be used in analysis. Data from the follow-up sample show results from baseline and follow-up protocol administrations. Study 3 protocol not included at follow-up. ** At least one message was helpful for quitting; therefore data could be used in analysis. Data from the follow-up sample show results from baseline and follow-up protocol administrations. HIS—Heaviness of smoking index; DCE—Discrete choice experiment.

**Table 2:** Effects of cessation benefit message characteristics on quit motivation.

Characteristics		Baseline DCE 1	Follow-Up DCE 1
		*b* (SE)	*b* (SE)
Model 1: Main effects of message characteristics			
Message Topic			
improving lung health		0.000 (0.004)	−0.008 (0.006)
avoiding diabetes		0.014 ** (0.004)	0.015 * (0.006)
new disease		0.015 *** (0.004)	0.007 (0.006)
enhancing wellbeing		−0.013 ** (0.004)	0.011 (0.006)
financial benefits		−0.016 *** (0.004)	−0.025 *** (0.006)
Overall *p*-value		<0.001	<0.001
Imagery Type			
young man		−0.007 (0.004)	0.001 (0.006)
young woman		0.008 (0.004)	0.009 (0.006)
older man		−0.005 (0.004)	−0.009 (0.006)
older woman		−0.001 (0.004)	0.001 (0.006)
symbolic		0.005 (0.004)	−0.003 (0.006)
Overall *p*-value		0.150	0.436
Model 2: Participant sex X Image type			
Male participant	young man	−0.007 (0.007)	0.011 (0.010)
	young woman	0.013 (0.007)	−0.004 (0.009)
	older man	−0.006 (0.007)	0.000 (0.009)
	older woman	−0.015 * (0.007)	−0.003 (0.010)
	symbolic	0.016 * (0.007)	0.004 (0.009)
Female participant	young man	−0.007 (0.006)	−0.007 (0.009)
	young woman	0.003 (0.006)	0.020 * (0.009)
	older man	−0.005 (0.006)	−0.016 (0.009)
	older woman	0.009 (0.006)	0.004 (0.009)
	symbolic	−0.002 (0.006)	−0.008 (0.009)
Overall *p*-value		0.052	0.242
Model 3: Participant age X Image type			
18–39 years old participant	young man	−0.003 (0.007)	0.008 (0.011)
	young woman	0.001 (0.007)	0.014 (0.010)
	older man	0.005 (0.007)	−0.013 (0.011)
	older woman	−0.002 (0.007)	0.004 (0.011)
	symbolic	0.005 (0.007)	0.000 (0.011)
40–64 years old participant	young man	−0.010 (0.006)	−0.003 (0.008)
	young woman	0.013 * (0.006)	0.005 (0.008)
	older man	−0.014 * (0.006)	−0.008 (0.008)
	older woman	−0.001 (0.006)	−0.001 (0.008)
	symbolic	0.006 (0.006)	−0.006 (0.008)
Overall *p*-value		0.190	0.820

* *p* < 0.05; ** *p* < 0.01; *** *p* < 0.001.

**Table 3:** Effects of quitting tips message characteristics on helpfulness for quitting.

Characteristics		Baseline DCE 2	Follow-Up DCE 2
		*b* (SE)	*b* (SE)
Model 1: Main effects of message characteristics			
Message Topic			
stress reduction		0.013 ** (0.004)	0.002 (0.006)
list of cessation strategies		−0.046 *** (0.004)	−0.032 *** (0.006)
nicotine replacement therapy		0.011 * (0.004)	0.010 (0.006)
social support		0.017 *** (0.005)	0.015 * (0.006)
physical activity		0.005 (0.004)	0.005 (0.006)
Overall *p*-value		<0.001	<0.001
Imagery Type			
young man		−0.006 (0.004)	0.002 (0.006)
young woman		0.003 (0.004)	−0.005 (0.006)
older man		0.003 (0.004)	0.004 (0.006)
older woman		−0.005 (0.004)	0.006 (0.006)
symbolic		0.005 (0.004)	−0.007 (0.006)
Overall *p*-value		0.375	0.546
Model 2: Participant sex X Image type			
Male participant	young man	−0.001 (0.007)	0.009 (0.009)
	young woman	0.006 (0.007)	−0.010 (0.010)
	older man	0.006 (0.007)	0.019 * (0.010)
	older woman	−0.013 (0.007)	−0.020 * (0.010)
	symbolic	0.005 (0.007)	−0.003 (0.009)
Female participant	young man	−0.010 (0.006)	−0.003 (0.009)
	young woman	0.000 (0.006)	−0.001 (0.009)
	older man	0.001 (0.006)	−0.009 (0.009)
	older woman	0.001 (0.006)	0.029 ** (0.009)
	symbolic	0.004 (0.006)	−0.011 (0.009)
Overall *p*-value		0.584	0.010
Model 3: Participant age X Image type			
18–39 years old participant	young man	0.003 (0.007)	0.012 (0.011)
	young woman	−0.002 (0.007)	−0.012 (0.011)
	older man	0.000 (0.007)	−0.010 (0.011)
	older woman	−0.001 (0.007)	0.010 (0.011)
	symbolic	0.005 (0.007)	0.001 (0.011)
40–64 years old participant	young man	−0.014 * (0.006)	−0.003 (0.008)
	young woman	0.006 (0.006)	−0.002 (0.008)
	older man	0.006 (0.006)	0.012 (0.008)
	older woman	−0.008 (0.006)	0.003 (0.008)
	symbolic	0.004 (0.006)	−0.012 (0.008)
Overall *p*-value		0.447	0.507

* *p* < 0.05; ** *p* < 0.01; *** *p* < 0.001.

**Table 4:** Effects of reproductive health benefit message characteristics on quit motivation.

Characteristics		Baseline DCE 3
		*b* (SE)
Model 1: Main effects of message characteristics		
Message Topic		
health of mom & baby		0.093 *** (0.012)
health of mom & baby + quit tips		−0.006 (0.013)
health of mom, dad & baby		0.031 * (0.013)
fertility of mom & dad, healthy pregnancy & baby		−0.118 *** (0.011)
Overall *p*-value		<0.001
Imagery Type		
pregnant mom		0.023 (0.013)
mom with baby		0.107 *** (0.011)
mom & dad with baby		0.122 *** (0.015)
symbolic (pregnant mom)		−0.252 *** (0.011)
Overall *p*-value		<0.001
Model 2: Participant sex X Image type		
Male participant	pregnant mom	−0.012 (0.020)
	mom with baby	0.092 *** (0.017)
	mom & dad with baby	0.223 *** (0.024)
	symbolic (pregnant mom)	−0.266 *** (0.017)
Female participant	pregnant mom	0.045 * (0.018)
	mom with baby	0.116 *** (0.015)
	mom & dad with baby	0.045 * (0.021)
	symbolic (pregnant mom)	−0.244 *** (0.019)
Overall *p*-value		<0.001
Model 3: Participant age X Image type		
18–39 years old participant	pregnant mom	0.032 (0.019)
	mom with baby	0.094 *** (0.016)
	mom & dad with baby	0.111 *** (0.022)
	symbolic (pregnant mom)	−0.240 *** (0.016)
40–64 years old participant	pregnant mom	0.013 (0.019)
	mom with baby	0.120 *** (0.016)
	mom & dad with baby	0.134 *** (0.023)
	symbolic (pregnant mom)	−0.264 *** (0.019)
Overall *p*-value		<0.001

* *p* < 0.05; *** *p* < 0.001.

## Supplementary Materials

The following are available online at http://www.mdpi.com/1660-4601/15/2/282/s1, Figure S1: Example choice set for DCE 2, self-efficacy messages that target general audiences of smokers, Figure S2: Example choice set for DCE 3, reproductive health response efficacy messages that target general audiences of smokers, Figure S3: Interactions between message topic and image type; Results from DCE 1, general response efficacy messages (*p* < 0.05 for contrast with the grand mean), Figure S4: Interactions between message topic and image type; Results from DCE 2, general self-efficacy messages (*p* < 0.05 for contrast with the grand mean), Figure S5: Interactions between message topic and image type; Results from DCE 3, reproductive health response efficacy messages (* *p* < 0.05 for contrast with the grand mean), Table S1: Choice sets and blocks for DCE 1, Response Efficacy, Table S2: Choice sets and blocks for DCE 2, Self-Efficacy, Table S3: Choice sets and blocks for DCE 3, Reproductive health response efficacy.

## References

[1] Canadian Cancer Society (2016). Cigarette Package Health Warnings: International Status Report.

[2] Noar S.M., Francis D.B., Bridges C., Sontag J.M., Ribisl K.M., Brewer N.T. (2016). The impact of strengthening cigarette pack warnings: Systematic review of longitudinal observational studies. Soc. Sci. Med..

[3] Noar S.M., Hall M.G., Francis D.B., Ribisl K.M., Pepper J.K., Brewer N.T. (2016). Pictorial cigarette pack warnings: A meta-analysis of experimental studies. Tob. Control.

[4] Popova L. (2014). Scaring the snus out of smokers: Testing effects of fear, threat, and efficacy on smokers’ acceptance of novel smokeless tobacco products. Health Commun..

[5] Witte K., Allen M. (2000). A meta-analysis of fear appeals: Implications for effective public health campaigns. Health Educ. Behav..

[6] Thrasher J.F., Osman A., Abad-Vivero E.N., Hammond D., Bansal-Travers M., Cummings K.M., Hardin J.V.V., Moodie C. (2015). The Use of Cigarette Package Inserts to Supplement Pictorial Health Warnings: An Evaluation of the Canadian Policy. Nicotine Tob. Res..

[7] Thrasher J.F., Swayampakala K., Cummings K.M., Hammond D., Anshari D., Krugman D.M., Hardin J.W. (2016). Cigarette package inserts can promote efficacy beliefs and sustained smoking cessation attempts: A longitudinal assessment of an innovative policy in Canada. Prev. Med..

[8] Thrasher J.F., Carpenter M.J., Andrews J.O., Gray K.M., Alberg A.J., Navarro A., Friedman D.B., Cumminqs K.M. (2012). Cigarette warning label policy alternatives and smoking-related health disparities. Am. J. Prev. Med..

[9] Hammond D., Reid J.L., Driezen P., Boudreau C. (2013). Pictorial health warnings on cigarette packs in the United States: An experimental evaluation of the proposed FDA warnings. Nicotine Tob. Res..

[10] Hammond D., Thrasher J., Reid J.L., Driezen P., Boudreau C., Santillan E.A. (2012). Perceived effectiveness of pictorial health warnings among Mexican youth and adults: A population-level intervention with potential to reduce tobacco-related inequities. Cancer Causes Control.

[11] Thrasher J., Arillo-Santillán E., Villalobos V., Pérez-Hernández R., Hammond D., Carter J., Sebrié E., Sansores R., Regalado-Pineda J. (2012). Can pictorial warning labels on cigarette packages address smoking-related health disparities? Field experiments in Mexico to assess pictorial warning label content. Cancer Causes Control.

[12] Hammond D., Fong G.T., MacDonald P., Cameron R., Brown K. (2003). Impact of graphic Canadian warning labels on adult smoking behavior. Tob. Control.

[13] Thrasher J., Anshari D., Lambert V., Islam F., Mead E., Popova L., Salloum R.G., Moodie C., Louviere J., Lindblom E.N. (2018). Assessing smoking cessation messages with a discrete choice experiment. Tob. Regul. Sci..

[14] Terry-McElrath Y., Wakefield M., Ruel E., Balch G.I., Emery S., Szczypka G., Cleqq-Smith K., Flay B. (2005). The effect of antismoking advertisement executional characteristics on youth comprehension, appraisal, recall, and engagement. J. Health Commun..

[15] Zillmann D. (2006). Exemplification effects in the promotion of safety and health. J. Commun..

[16] Petty R.E., Cacioppo J.T. (1986). The elaboration likelihood model of persuasion. Adv. Exp. Soc. Psychol..

[17] Brewer N., Hall M., Noar S., Parada H., Stein-Seroussi A., Bach L.E., Hanley S., Ribisl K.M. (2016). Effect of pictorial cigarette pack warnings on changes in smoking behavior: A randomized clinical trial. JAMA Intern. Med..

[18] Evans A., Peters E., Strasser A., Emery L., Sheerin K., Romer D. (2015). Graphic warning labels elicit affective and thoughtful responses from smokers: Results of a randomized clinical trial. PLoS ONE.

[19] McQueen A., Kreuter M., Boyum S., Thompson V., Caburnay C., Waters E., Kaphinqst K.A., Rath S., Fu Q. (2015). Reactions to FDA-proposed graphic warning labels affixed to US smokers’ cigarette packs. Nicotine Tob. Res..

[20] Mutti-Packer S., Reid J.L., Thrasher J.F., Romer D., Fong G.T., Gupta P.C., Pednekar M.S., Narqis N., Hammond D. (2017). The role of negative affect and message credibility in perceived effectiveness of smokeless tobacco health warning labels in Navi Mumbai, India and Dhaka, Bangladesh: A moderated-mediation analysis. Addict. Behav..

[21] Cho Y., Thrasher J., Yong H.-H., Szklo A., O’Connor R., Bansal-Travers M., Hammond D., Fong G.T., Hardin J., Borland R. (2018). Path analysis of warning label effects on negative emotions and quit attempts: A longitudinal study of smokers in Australia, Canada, Mexico, and the US. Soc. Sci. Med..

[22] Thrasher J., Villalobos V., Szklo A., Fong G.T., Pérez C., Sebrié E.M., Sansone N., Fiqueiredo V., Boado M., Arillo-Santillan E. (2010). Assessing the impact of cigarette package warning labels: A cross-country comparison in Brazil, Uruguay and Mexico. Salud Pública Méx..

[23] World Health Organization (WHO) (2014). How Large Pictorial Health Warnings on the Packaging of Tobacco Products Affect Knowledge and Behaviour.

[24] Kreuter M.W., Strecher V.J., Glassman B. (1999). One size does not fit all: The case for tailoring print materials. Ann. Behav. Med..

[25] McGuire W.J., Rice R.E., Atkin C.K. (1991). Theoretical foundations of campaigns. Public Communication Campaigns.

[26] Hawkins R.P., Kreuter M., Resnicow K., Fishbein M., Dijkstra A. (2008). Understanding tailoring in communicating about health. Health Educ. Res..

[27] Skinner C.S., Campbell M.K., Rimer B.K., Curry S., Prochaska J.O. (1999). How effective is tailored print communication?. Ann. Behav. Med..

[28] Kollath-Cattano C., Osman A., Thrasher J.F. (2017). Evaluating the perceived effectiveness of pregnancy-related cigarette package health warning labels among different gender/age groups. Addict. Behav..

[29] Osman A., Thrasher J., Yong H.-H., Arillo-Santillán E., Hammond D. (2017). Disparagement of health warning labels on cigarette packages and smoking cessation: Results from four countries. Health Educ. Res..

[30] Thrasher J., Osman A., Moodie C., Hammond D., Bansal-Travers M., Cummings K., Borland R., Yong H.H., Hardin J. (2015). Promoting cessation resources through cigarette package warning labels: A longitudinal survey with adult smokers in Australia, Canada and Mexico. Tob. Control.

[31] Willemsen M.C. (2005). The new EU cigarette health warnings benefit smokers who want to quit the habit: Results from the Dutch continuous survey of smoking habits. Eur. J. Public Health.

[32] Villanti A., Cantrell J., Pearson J., Vallone D., Rath J. (2014). Perceptions and perceived impact of graphic cigarette health warning labels on smoking behavior among U.S. young adults. Nicotine Tob. Res..

[33] Clark M.D., Determann D., Petrou S., Moro D., de Bekker-Grob E.W. (2014). Discrete choice experiments in health economics: A review of the literature. Pharmacoeconomics.

[34] De Bekker-Grob E.W., Ryan M., Gerard K. (2012). Discrete choice experiments in health economics: A review of the literature. Health Econ..

[35] Lancsar E., Louviere J. (2008). Conducting discrete choice experiments to inform healthcare decision making: A user’s guide. Pharmacoeconomics.

[36] Louviere J., Hensher D., Swait J. (2000). Stated Choice Methods: Analysis and Applications.

[37] Phillip Morris (1998). Coupon Optimization at Individual Level Conjoint Analysis.

[38] Dennis & Company Research (1999). Cigarette Visual Comparison Study: Concept Evaluation.

[39] Hirji T. (1996). Project Sky Conjoint.

[40] Devinney T. (2014). Analysis of Consumer Research Evidence on the Impact of Plain Packaging for Tobacco Products (Updated to 2014).

[41] Hoek J., Gendall P., Eckert C., Louviere J. (2016). Dissuasive cigarette sticks: The next step in standardised (‘plain’) packaging?. Tob. Control.

[42] Kotnowski K., Fong G.T., Gallopel-Morvan K., Islam T., Hammond D. (2016). The impact of cigarette packaging design among young females in Canada: Findings from a discrete choice experiment. Nicotine Tob. Res..

[43] Hoek J., Gendall P., Eckert C., Kemper J., Louviere J. (2016). Effects of brand variants on smokers’ choice behaviours and risk perceptions. Tob. Control.

[44] Salloum R.G., Louviere J.J., Getz K.R., Islam F., Anshari D., Cho Y., O’Connor R.J., Hammond D., Thrasher J.F. (2017). Evaluation of strategies to communicate harmful and potentially harmful constituent (HPHC) information through cigarette package inserts: A discrete choice experiment. Tob. Control.

[45] Louviere J.J., Flynn T.N., Marley A.A. (2015). Best-Worst Scaling: Theory, Methods and Applications.

[46] Jackson S.A. (1992). Message Effects Research: Principles of Design and Analysis.

[47] Heatherton T.F., Kozlowski L.T., Frecker R.C., Rickert W., Robinson J. (1989). Measuring the Heaviness of Smoking: Using self-reported time to the first cigarette of the day and number of cigarettes smoked per day. Addiction.

[48] International Agency for Research on Cancer (IARC) (2009). IARC Handbooks of Cancer Prevention: Tobacco Control.

[49] Godin G., Kok G. (1996). The theory of planned behavior: A review of its applications to health-related behaviors. Am. J. Health Promot..

[50] Armitage C.J., Conner M. (2001). Efficacy of the Theory of Planned Behavior: A meta-analytic review. Br. J. Soc. Psychol..

[51] West R., Sohal T. (2006). “Catastrophic” pathways to smoking cessation: Findings from national survey. Br. Med. J..

[52] Viswanath K., Thomson G., Mitchell F., Williams M. (2006). Public communications and its role in reducing and eliminating health disparities. Examining the Health Disparities Research Plan of the National Institutes of Health: Unfinished Business.

[53] Cantrell J., Vallone D.M., Thrasher J., Nagler R.H., Feirman S.P., Muenz L.R., He D.Y., Viswanath K. (2013). Impact of tobacco-related health warning labels across socioeconomic, race and ethnic groups: Resutls from a randomized web-based experiment. PLoS ONE.

[54] Thrasher J., Rousu M.C., Hammond D., Navarro A.M., Corrigan J.R. (2011). Estimating the impact of pictorial health warnings and “plain” cigarette packaging: Evidence from experimental auctions among adult smokers in the United States. Health Policy.

[55] Gibson L., Brennan E., Momjian A., Shapiro-Luft D., Seitz H., Cappella J.N. (2015). Assessing the consequences of implementing graphic warning labels on cigarette packs for tobacco-related health disparities. Nicotine Tob. Res..

[56] Durkin S.J., Biener L., Wakefield M.A. (2009). Effects of different types of antismoking ads on reducing disparities in smoking cessation among socioeconomic subgroups. Am. J. Public Health.

[57] Niederdeppe J., Farrelly M.C., Nonnemaker J., Davis K.C., Wagner L. (2011). Socioeconomic variation in recall and perceived effectiveness of campaign advertisements to promote smoking cessation. Soc. Sci. Med..

[58] Niederdeppe J., Fiore M.C., Baker T.B., Smith S.S. (2008). Smoking-cessation media campaigns and their effectiveness among socioeconomically advantaged and disadvantaged populations. Am. J. Public Health.

[59] Lochbuehler K., Mercincavage M., Tang K., Tomlin D., Cappella J., Strasser A. (2017). Effect of message congruency on attention and recall in pictorial health warning labels. Tob. Control.

[60] Feinstein A., Cicchetti D. (1990). High agreement but low kappa: I. The problems of two paradoxes. J. Clin. Epidemiol..

[61] Huang L., Thrasher J., Reid J., Hammond D. (2016). Predictive and external validity of a pre-market study to determine the most effective pictorial health warning label content for cigarette packages. Nicotine Tob. Res..

[62] Osman A., Thrasher J., Cayir E., Hardin J., Perez-Hernandez R., Froeliger B. (2016). Depressive symptoms and responses to cigarette pack warning labels among Mexican smokers. Health Psychol..

