# Supplementary material for: Testing Cessation Messages for Cigarette Package Inserts: Findings from a Best/Worst Discrete Choice Experiment
Source: Int J Environ Res Public Health. 2018 Feb 6;15(2):282. doi: 10.3390/ijerph15020282 (PMC5858351; doi:10.3390/ijerph15020282)
Supplement: Supplementary file 1 [file ijerph-15-00282-s001.pdf]

Supplementary Materials

If you decided to quit, which message would be **MOST helpful** and which one would be **LEAST helpful** for increasing your confidence that you can successfully quit?

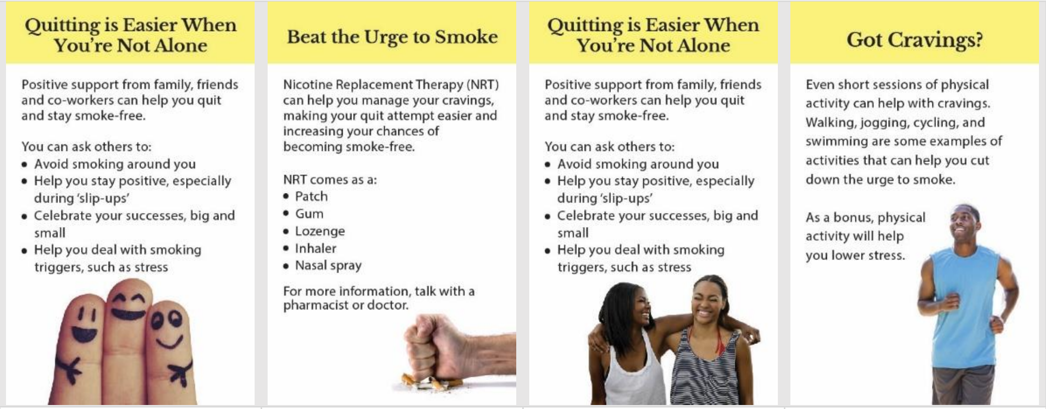

**Most** helpful	○	○	○	○
**Least** helpful	○	○	○	○

Thinking about these messages, do you actually think that:

Select one

**None** would be helpful if you decided to quit
**At least one** would be helpful if you decided to quit

Figure S1. Example choice set for DCE 2, self-efficacy messages that target general audiences of smokers.

Which message would **MOST motivate you** and which one would **LEAST motivate you to quit smoking**?

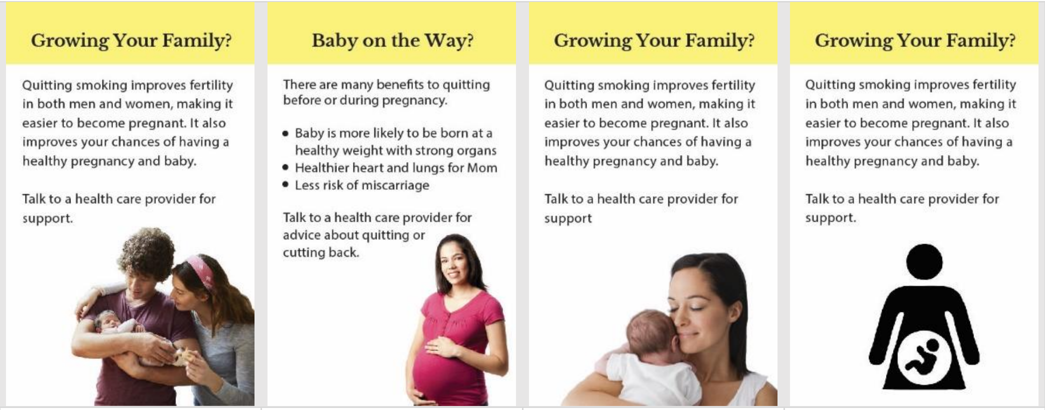

**Most** motivating	○	○	○	○
**Least** motivating	○	○	○	○

Thinking about these messages, do you actually think that:

Select one

**None** would motivate you to quit
**At least one** would motivate you to quit

Figure S2. Example choice set for DCE 3, reproductive health response efficacy messages that target general audiences of smokers.

Table S1. Choice sets and blocks for DCE 1; Response Efficacy.

Block	Set	Option 1	Option 2	Option 3	Option 4
1	1	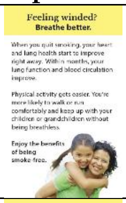	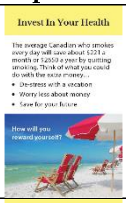	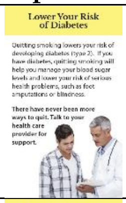	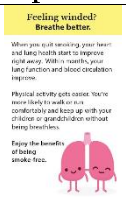
1	2	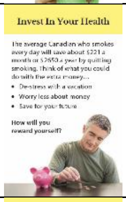	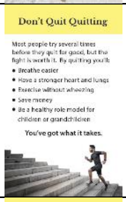	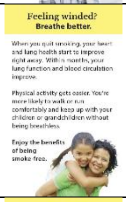	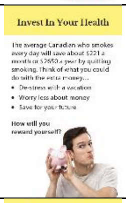
1	3	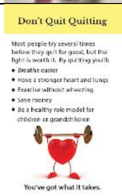	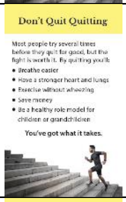	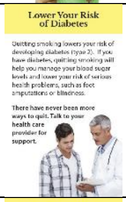	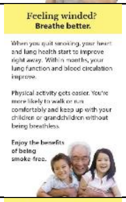
1	4	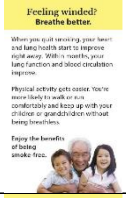	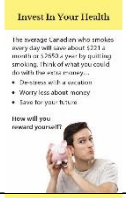	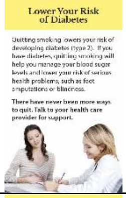	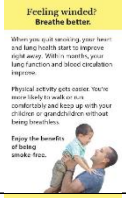
1	5	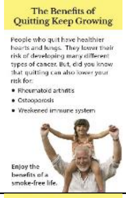	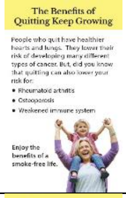	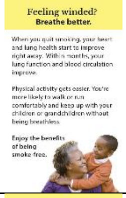	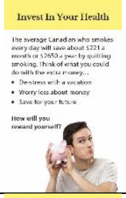
2	6	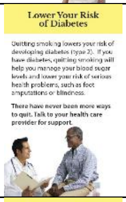	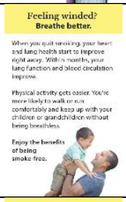	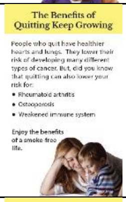	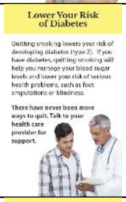
2	7	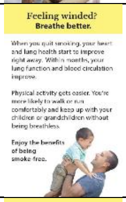	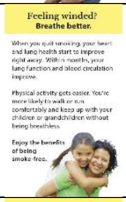	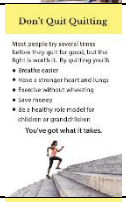	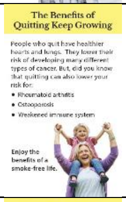
2	8	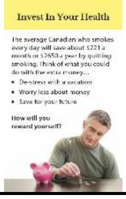	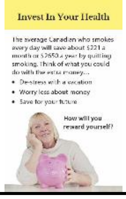	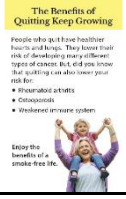	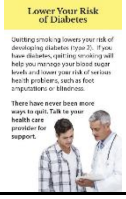

2	9	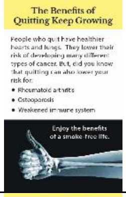	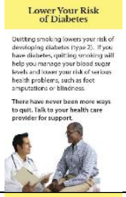	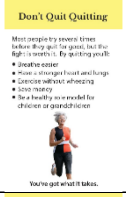	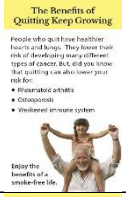
2	10	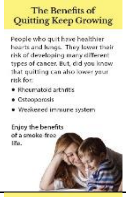	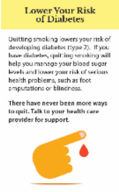	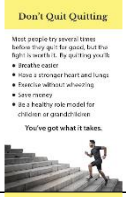	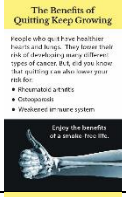
3	11	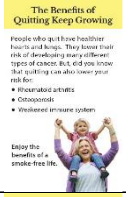	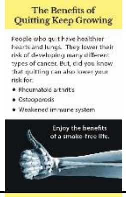	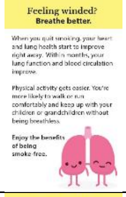	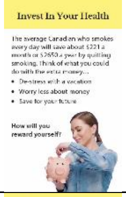
3	12	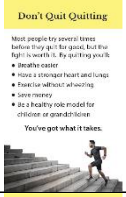	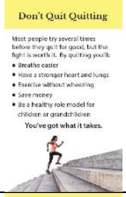	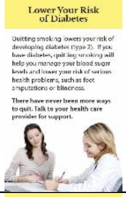	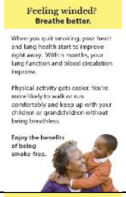
3	13	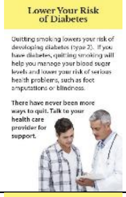	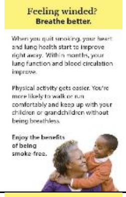	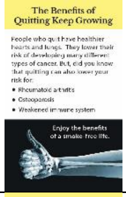	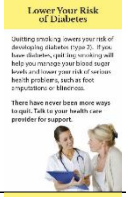
3	14	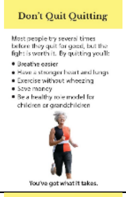	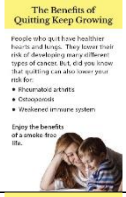	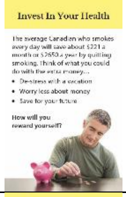	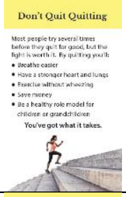
3	15	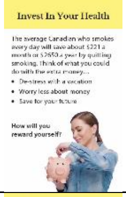	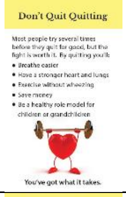	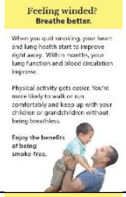	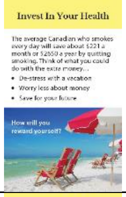
4	16	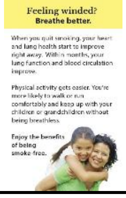	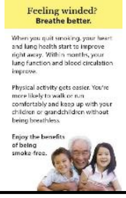	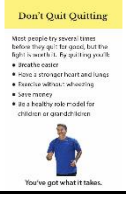	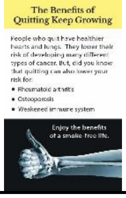

4	17	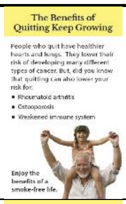	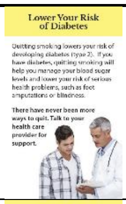	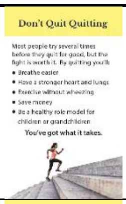	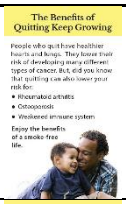
4	18	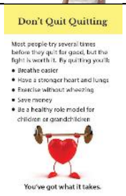	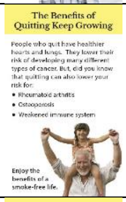	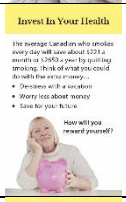	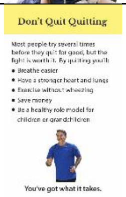
4	19	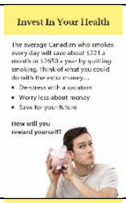	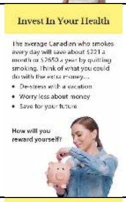	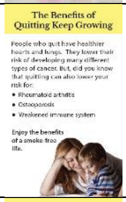	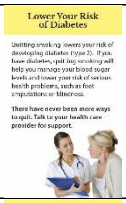
4	20	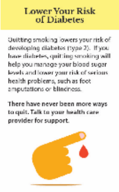	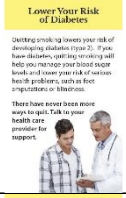	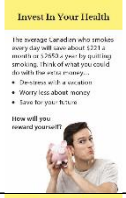	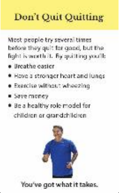
5	21	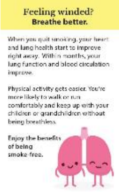	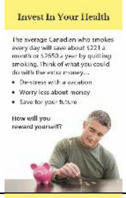	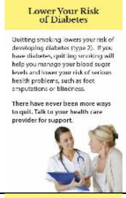	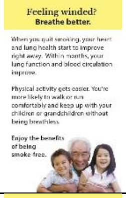
5	22	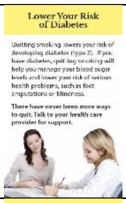	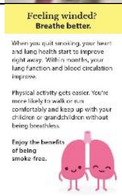	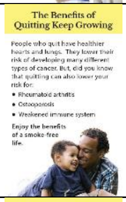	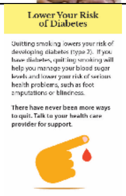
5	23	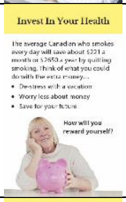	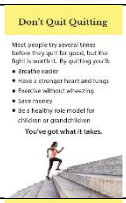	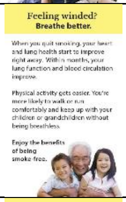	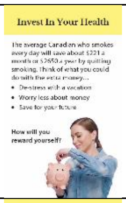
5	24	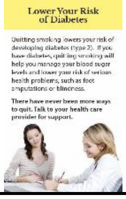	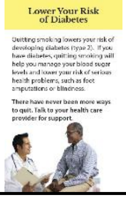	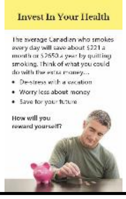	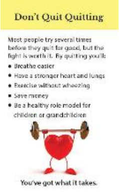

5	25	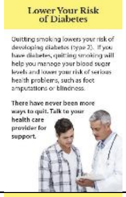	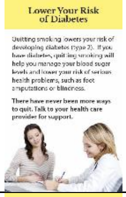	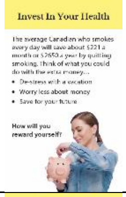	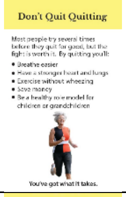
6	26	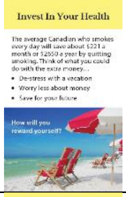	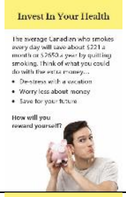	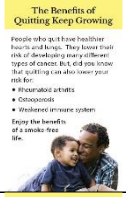	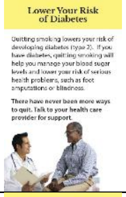
6	27	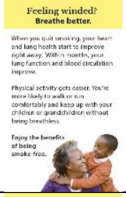	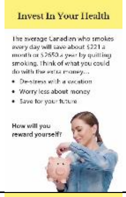	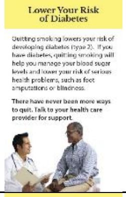	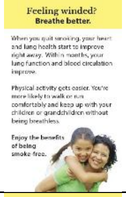
6	28	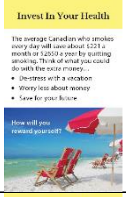	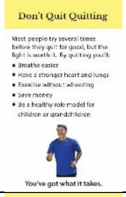	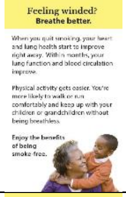	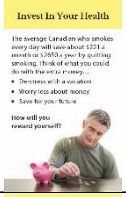
6	29	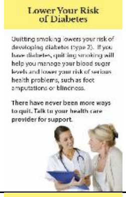	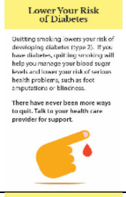	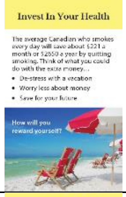	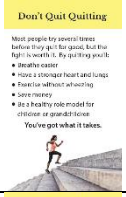
6	30	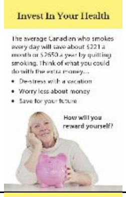	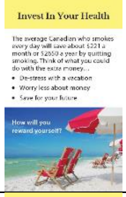	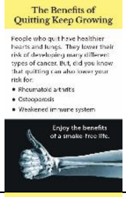	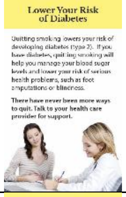
7	31	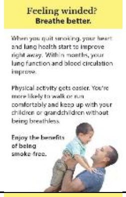	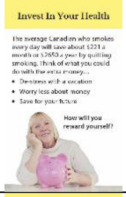	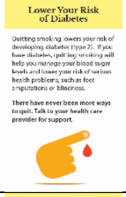	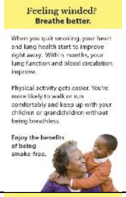
7	32	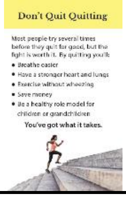	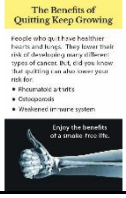	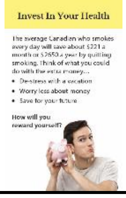	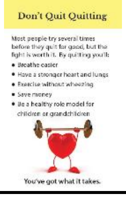

7	33	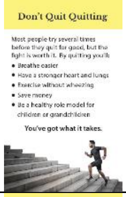	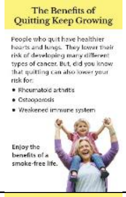	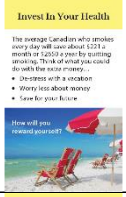	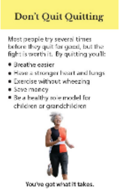
7	34	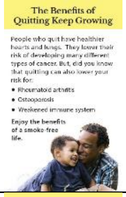	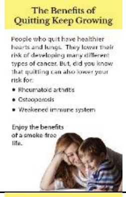	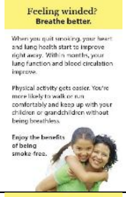	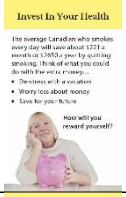
7	35	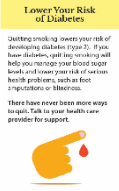	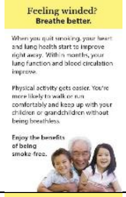	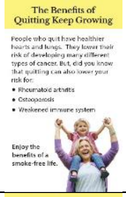	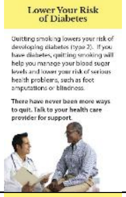
8	36	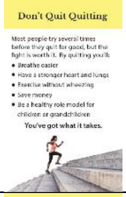	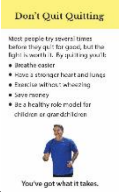	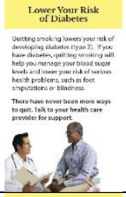	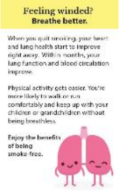
8	37	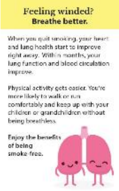	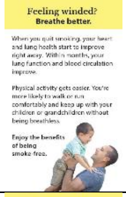	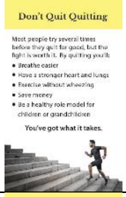	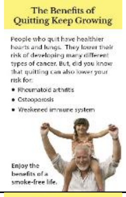
8	38	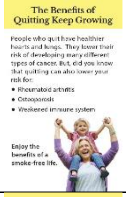	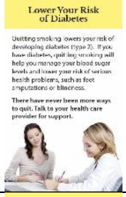	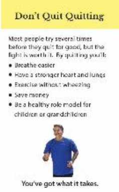	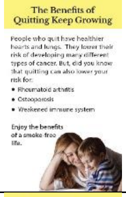
8	39	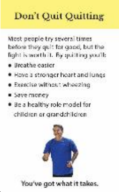	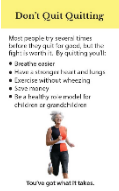	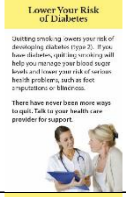	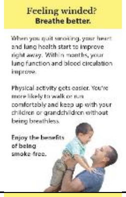
8	40	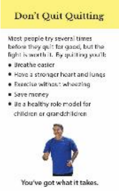	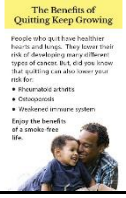	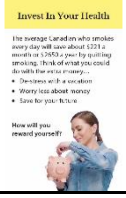	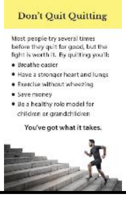

9	41	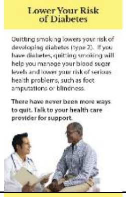	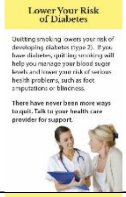	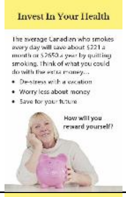	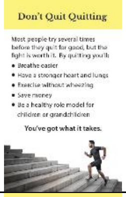
9	42	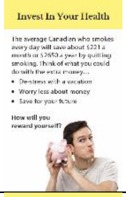	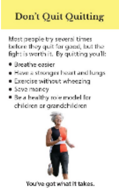	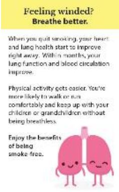	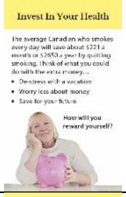
9	43	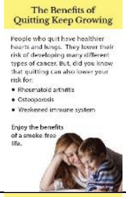	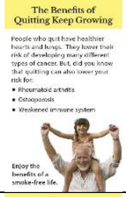	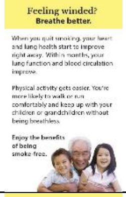	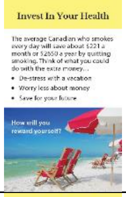
9	44	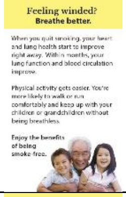	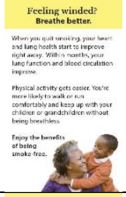	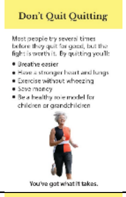	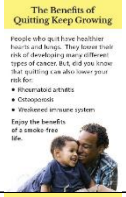
9	45	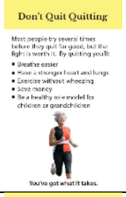	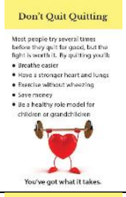	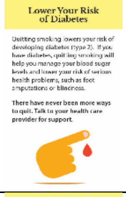	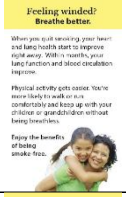
10	46	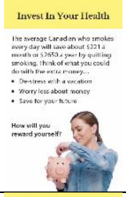	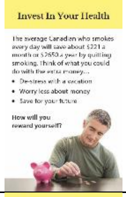	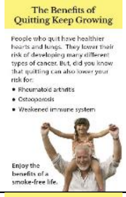	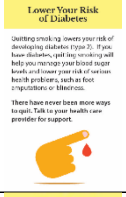
10	47	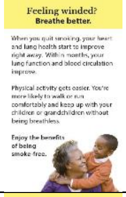	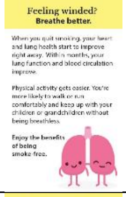	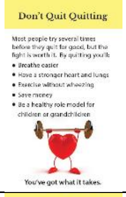	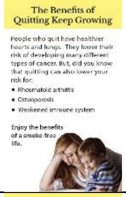
10	48	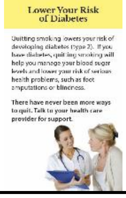	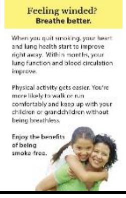	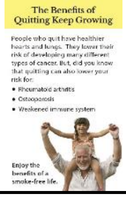	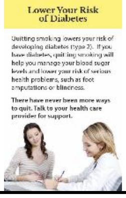

10	49	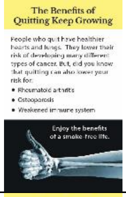The Benefits of Quitting Keep Growing<br>People who quit have healthier hearts and lungs. They lower their risk of developing many different types of cancer. But, did you know that quitting can also lower your risk for:<br>• Rheumatoid arthritis<br>• Osteoporosis<br>• Weakened immune system<br>Enjoy the benefits of a smoke-free life.	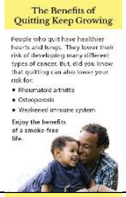The Benefits of Quitting Keep Growing<br>People who quit have healthier hearts and lungs. They lower their risk of developing many different types of cancer. But, did you know that quitting can also lower your risk for:<br>• Rheumatoid arthritis<br>• Osteoporosis<br>• Weakened immune system<br>Enjoy the benefits of a smoke-free life.	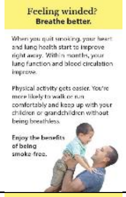Feeling winded?<br>Breathe better.<br>When you quit smoking, your heart and lung health start to improve right away. Within months, your lung function and blood circulation improve.<br>Physical activity gets easier. You're more likely to walk or run comfortably and keep up with your children or grandchildren without being breathless.<br>Enjoy the benefits of being smoke-free.	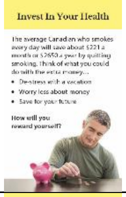Invest In Your Health<br>The average Canadian who smokes every day will save about $221 a month or $2650 a year by quitting smoking. Think of what you could do with the extra money…<br>• De-stress with a vacation<br>• Worry less about money<br>• Save for your future<br>How will you reward yourself?
10	50	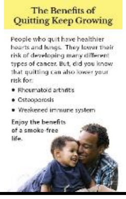The Benefits of Quitting Keep Growing<br>People who quit have healthier hearts and lungs. They lower their risk of developing many different types of cancer. But, did you know that quitting can also lower your risk for:<br>• Rheumatoid arthritis<br>• Osteoporosis<br>• Weakened immune system<br>Enjoy the benefits of a smoke-free life.	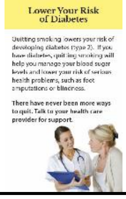Lower Your Risk of Diabetes<br>Quitting smoking lowers your risk of developing diabetes (type 2). If you have diabetes, quitting smoking will help you manage your blood sugar levels and lower your risk of serious health problems, such as foot amputations or blindness.<br>There have never been more ways to quit. Talk to your health care provider for support.	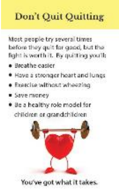Don't Quit Quitting<br>Most people try several times before they quit for good, but the fight is worth it. By quitting you'll:<br>• Breathe easier<br>• Have a stronger heart and lungs<br>• Exercise without wheezing<br>• Save money<br>• Be a healthy role model for children or grandchildren<br>You've got what it takes.	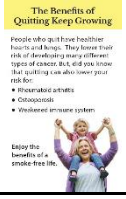The Benefits of Quitting Keep Growing<br>People who quit have healthier hearts and lungs. They lower their risk of developing many different types of cancer. But, did you know that quitting can also lower your risk for:<br>• Rheumatoid arthritis<br>• Osteoporosis<br>• Weakened immune system<br>Enjoy the benefits of a smoke-free life.

Table S2. Choice sets and blocks for DCE 2; Self-Efficacy.

Block	Set	Option 1	Option 2	Option 3	Option 4
1	1	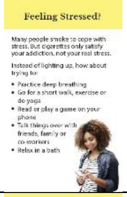	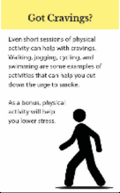	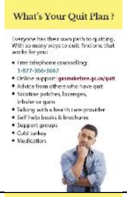	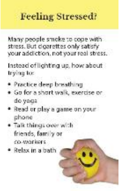
1	2	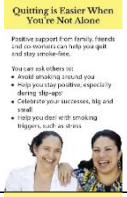	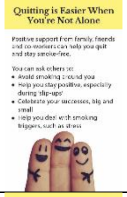	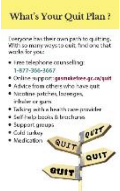	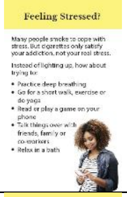
1	3	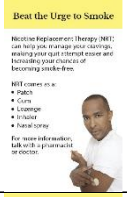	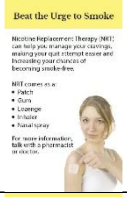	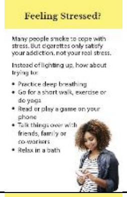	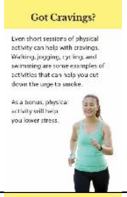
1	4	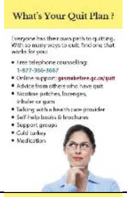	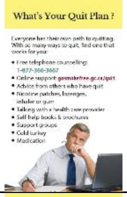	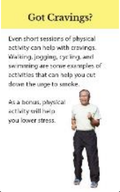	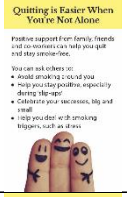
1	5	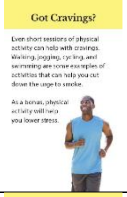	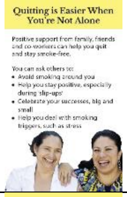	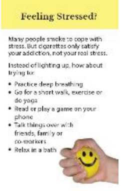	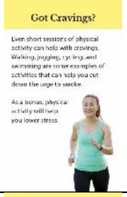
2	6	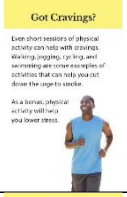	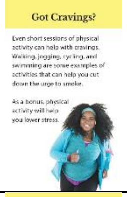	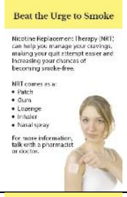	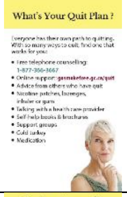
2	7	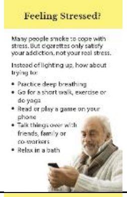	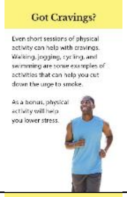	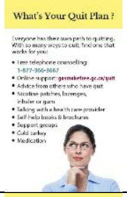	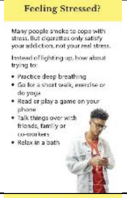
2	8	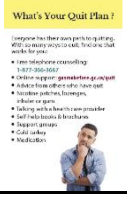	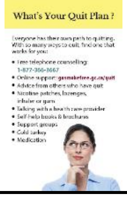	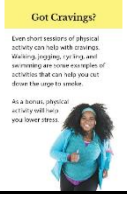	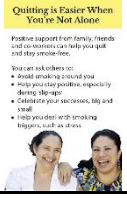

2	9	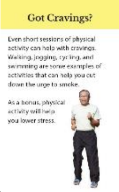	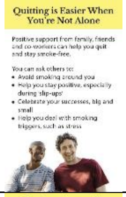	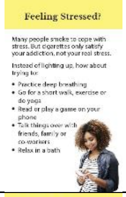	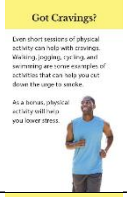
2	10	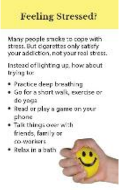	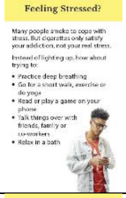	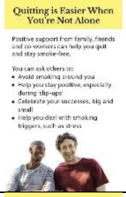	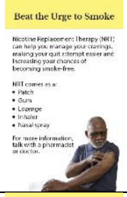
3	11	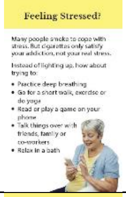	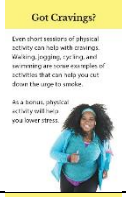	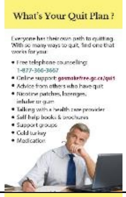	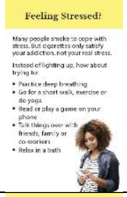
3	12	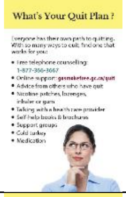	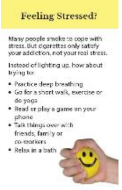	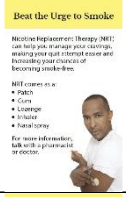	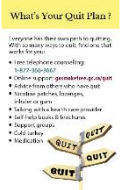
3	13	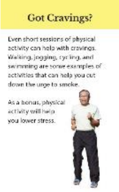	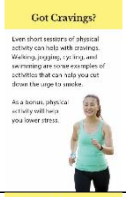	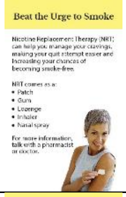	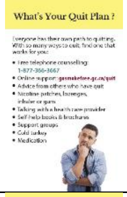
3	14	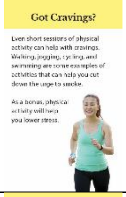	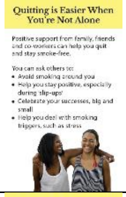	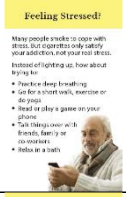	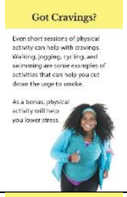
3	15	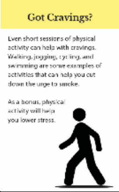	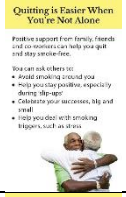	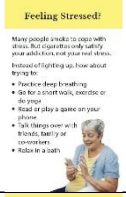	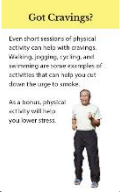
4	16	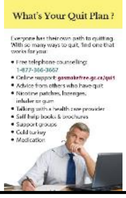	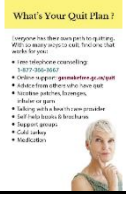	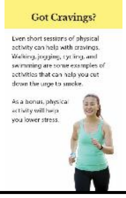	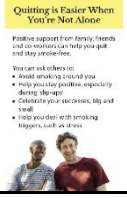

4	17	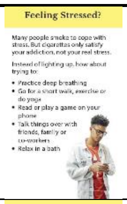	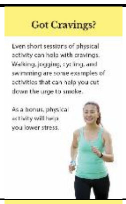	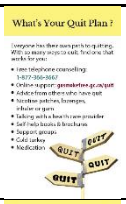	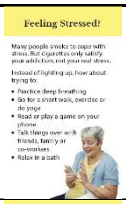
4	18	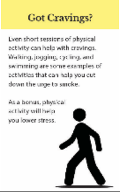	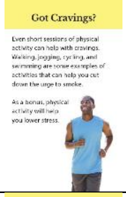	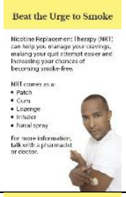	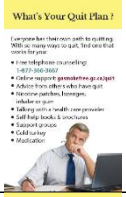
4	19	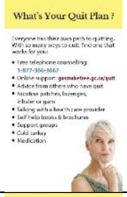	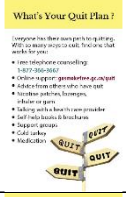	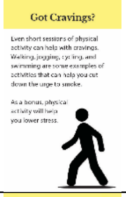	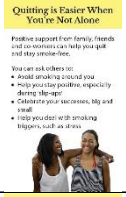
4	20	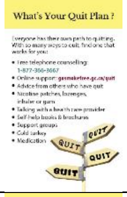	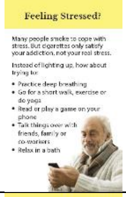	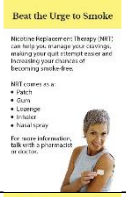	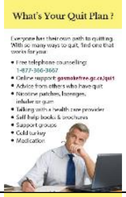
5	21	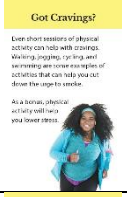	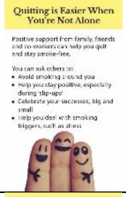	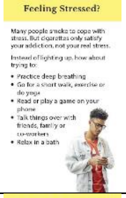	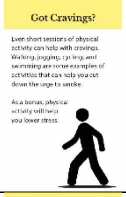
5	22	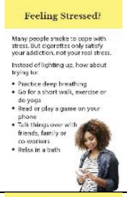	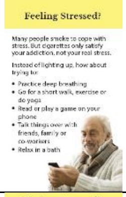	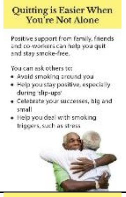	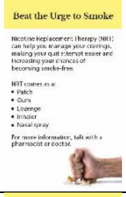
5	23	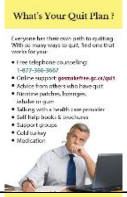	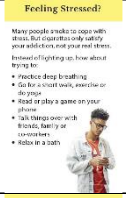	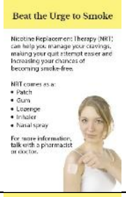	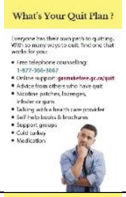
5	24	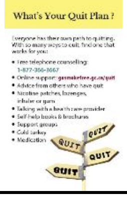	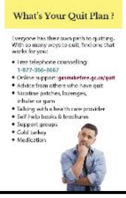	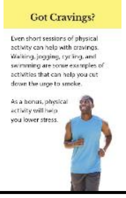	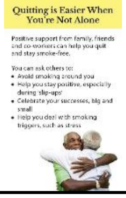

5	25	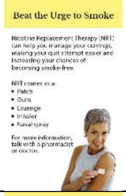	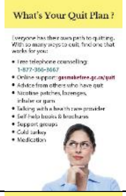	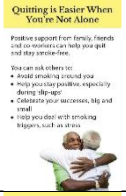	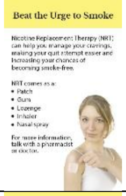
6	26	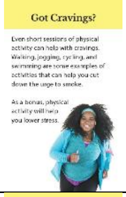	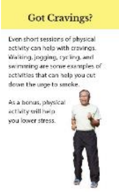	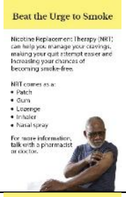	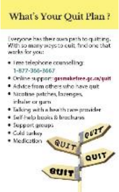
6	27	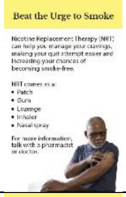	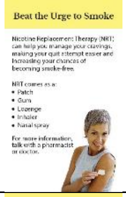	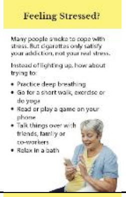	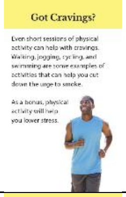
6	28	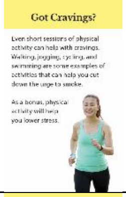	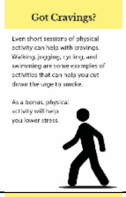	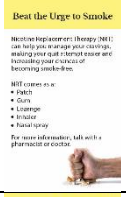	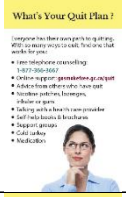
6	29	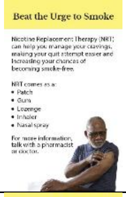	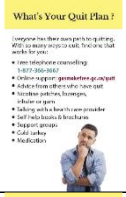	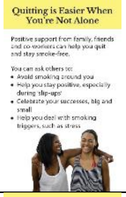	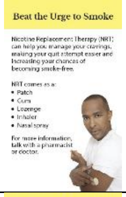
6	30	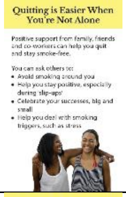	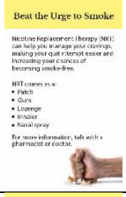	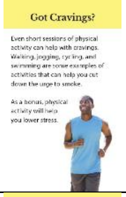	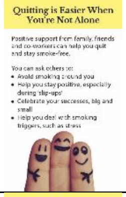
7	31	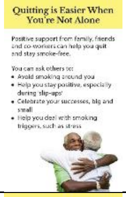	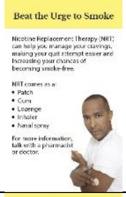	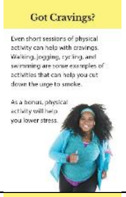	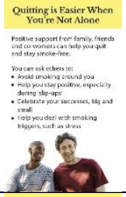
7	32	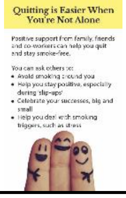	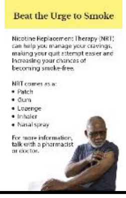	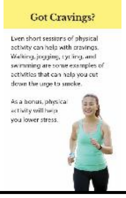	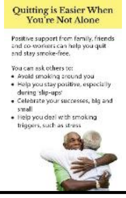

7	33	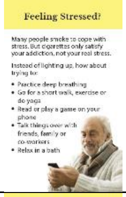	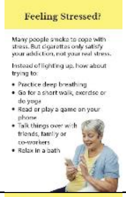	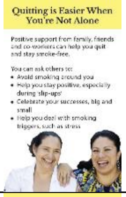	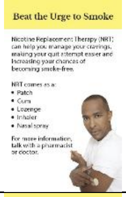
7	34	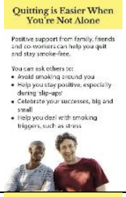	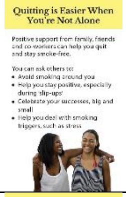	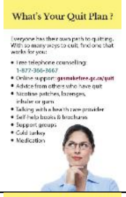	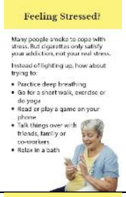
7	35	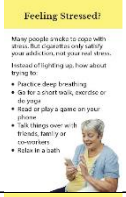	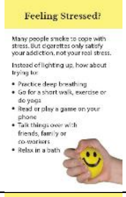	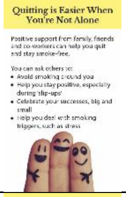	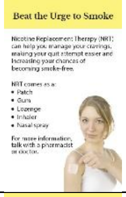
8	36	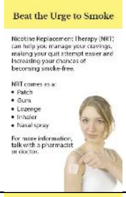	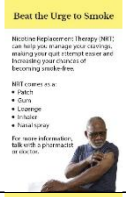	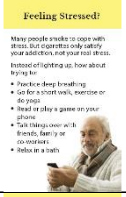	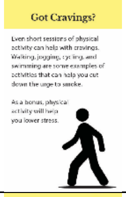
8	37	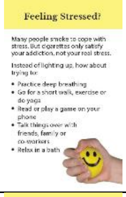	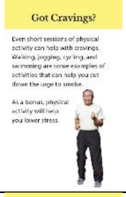	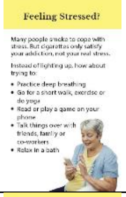	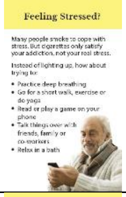
8	38	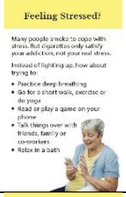	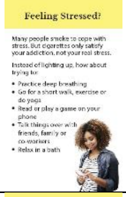	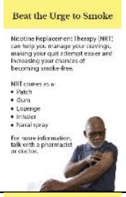	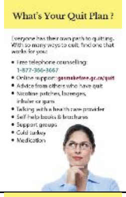
8	39	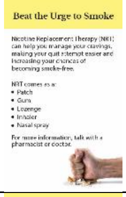	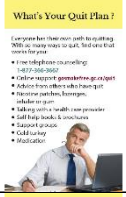	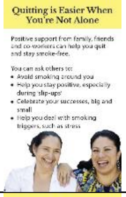	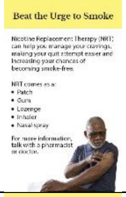
8	40	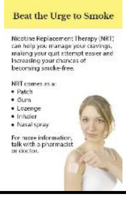	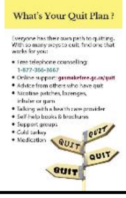	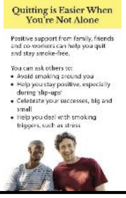	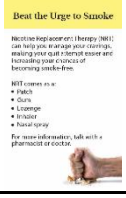

9	41	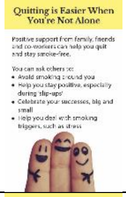	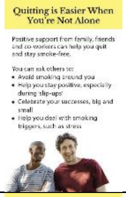	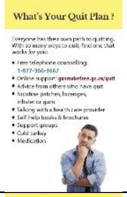	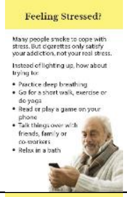
9	42	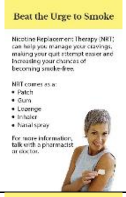	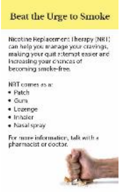	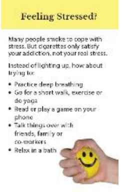	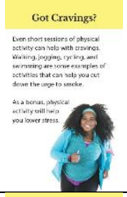
9	43	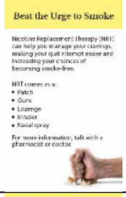	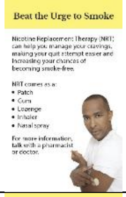	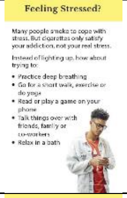	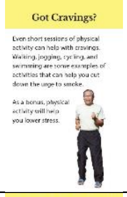
9	44	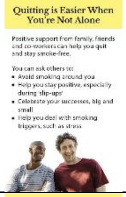	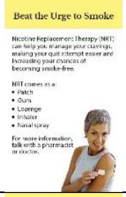	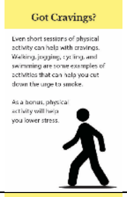	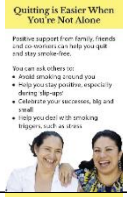
9	45	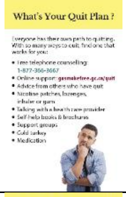	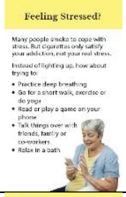	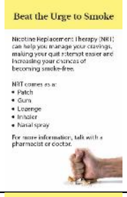	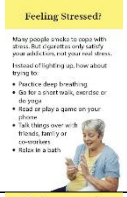
10	46	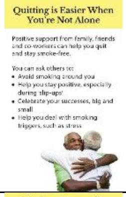	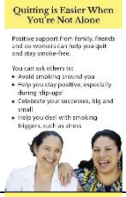	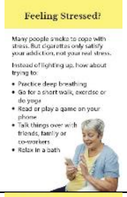	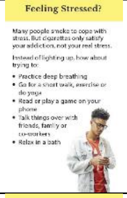
10	47	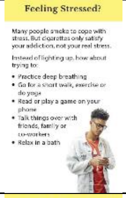	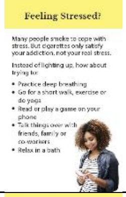	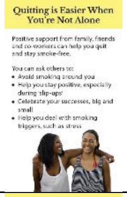	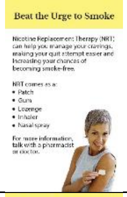
10	48	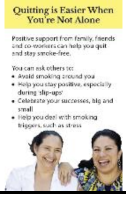	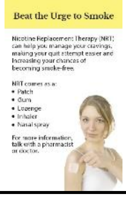	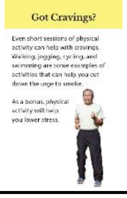	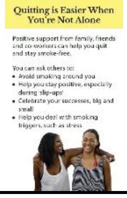

10	49	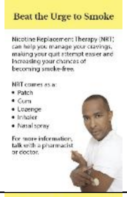Beat the Urge to Smoke	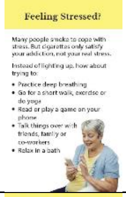Feeling Stressed?	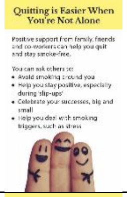Quitting is Easier When You're Not Alone	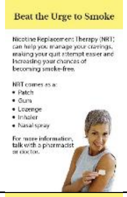Beat the Urge to Smoke
10	50	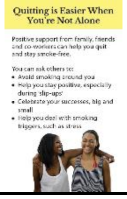Quitting is Easier When You're Not Alone	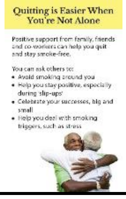Quitting is Easier When You're Not Alone	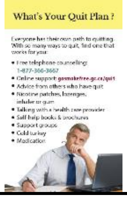What's Your Quit Plan ?	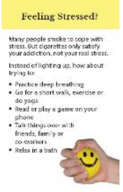Feeling Stressed?

Table S3. Choice sets and blocks for DCE 3; Reproductive health response efficacy.

Block	Set	Option 1	Option 2	Option 3	Option 4
1	1	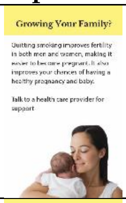	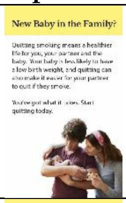	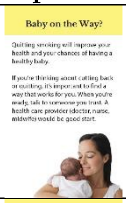	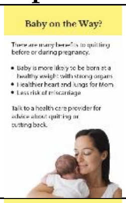
1	2	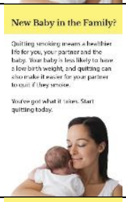	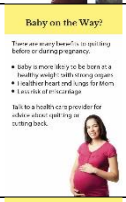	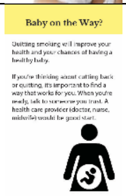	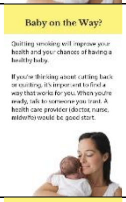
1	3	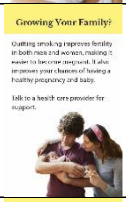	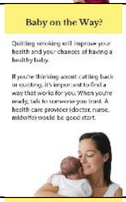	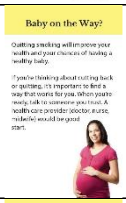	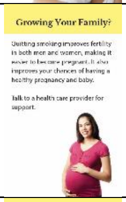
1	4	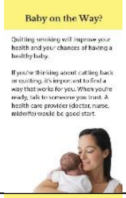	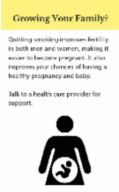	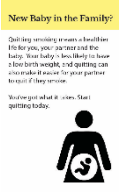	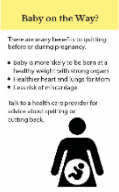
2	5	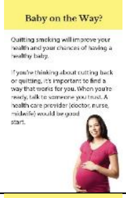	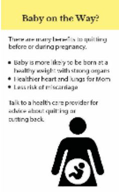	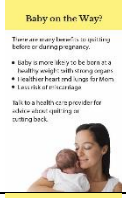	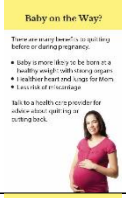
2	6	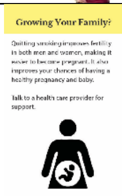	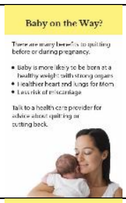	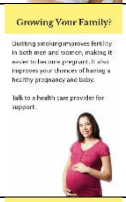	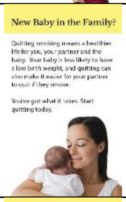
2	7	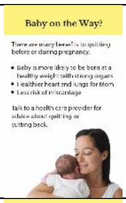	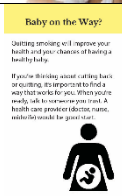	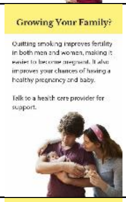	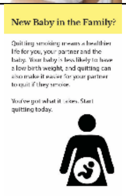
2	8	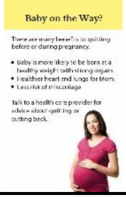	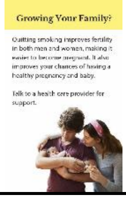	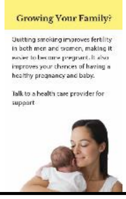	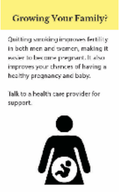

<table>
<tr><td>3</td><td>9</td><td>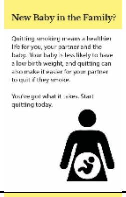
</td><td>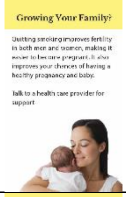
</td><td>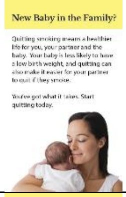
</td><td>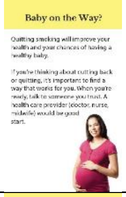
</td></tr>
<tr><td>3</td><td>10</td><td>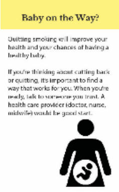
</td><td>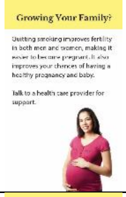
</td><td>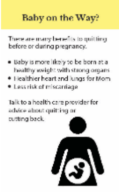
</td><td>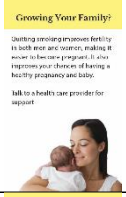
</td></tr>
<tr><td>3</td><td>11</td><td>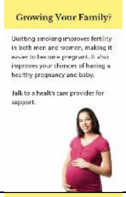
</td><td>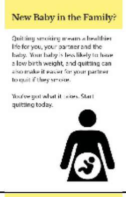
</td><td>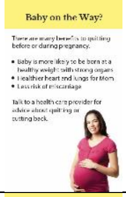
</td><td>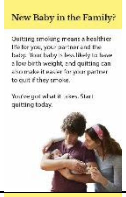
</td></tr>
<tr><td>3</td><td>12</td><td>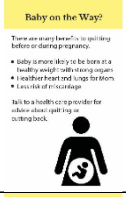
</td><td>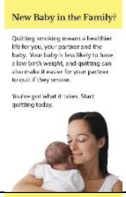
</td><td>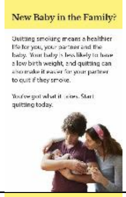
</td><td>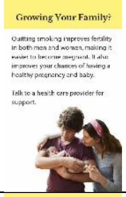
</td></tr>
<tr><td>3</td><td>13</td><td>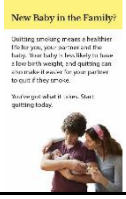
</td><td>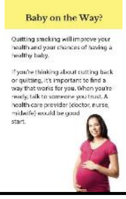
</td><td>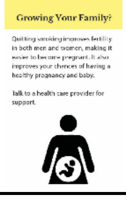
</td><td>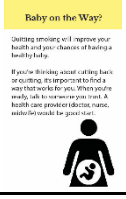
</td></tr>
</table>

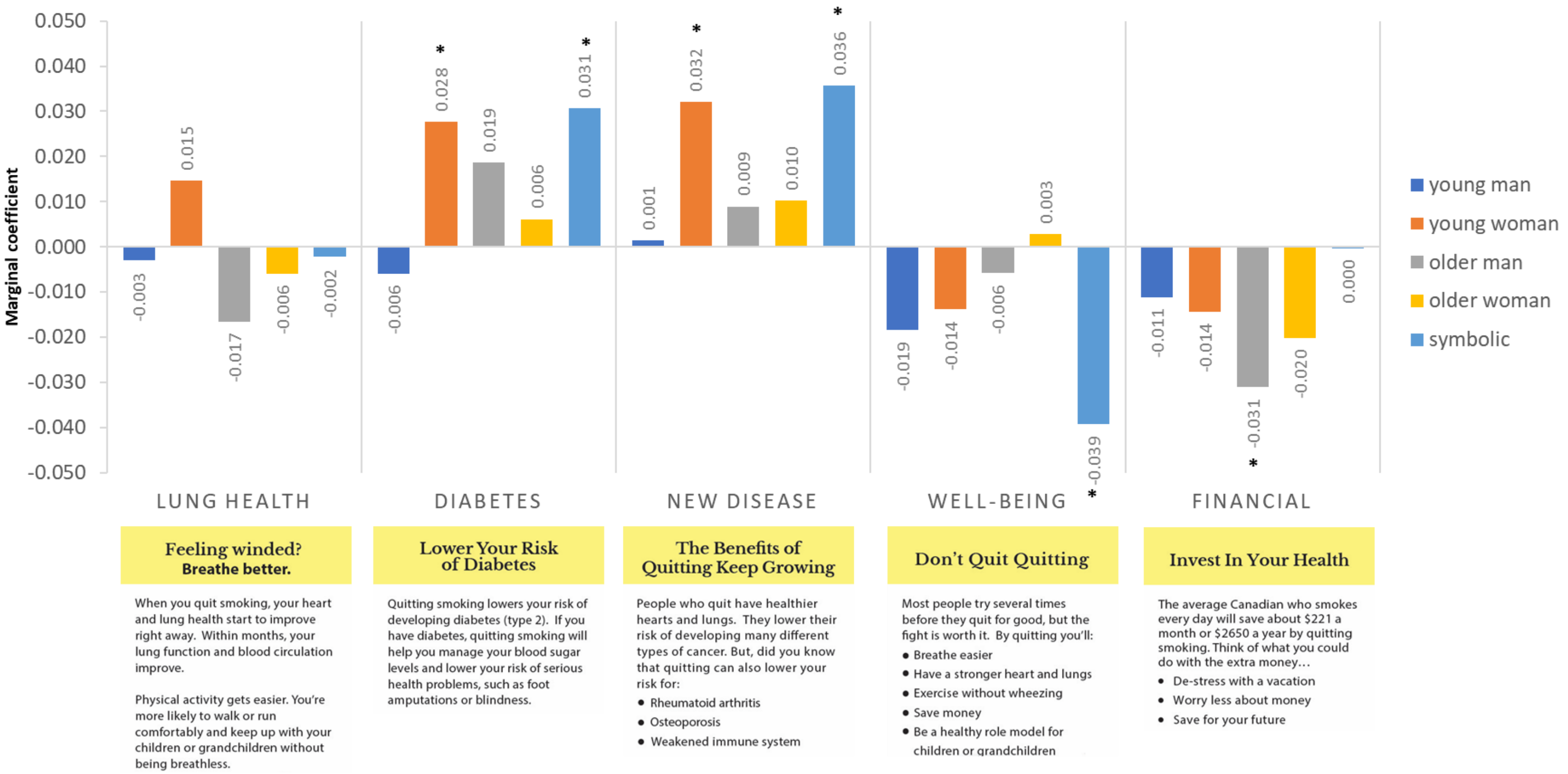

Figure S3. Interactions between message topic and image type; Results from DCE 1, general response efficacy messages (* $p < 0.05$ for contrast with the grand mean).

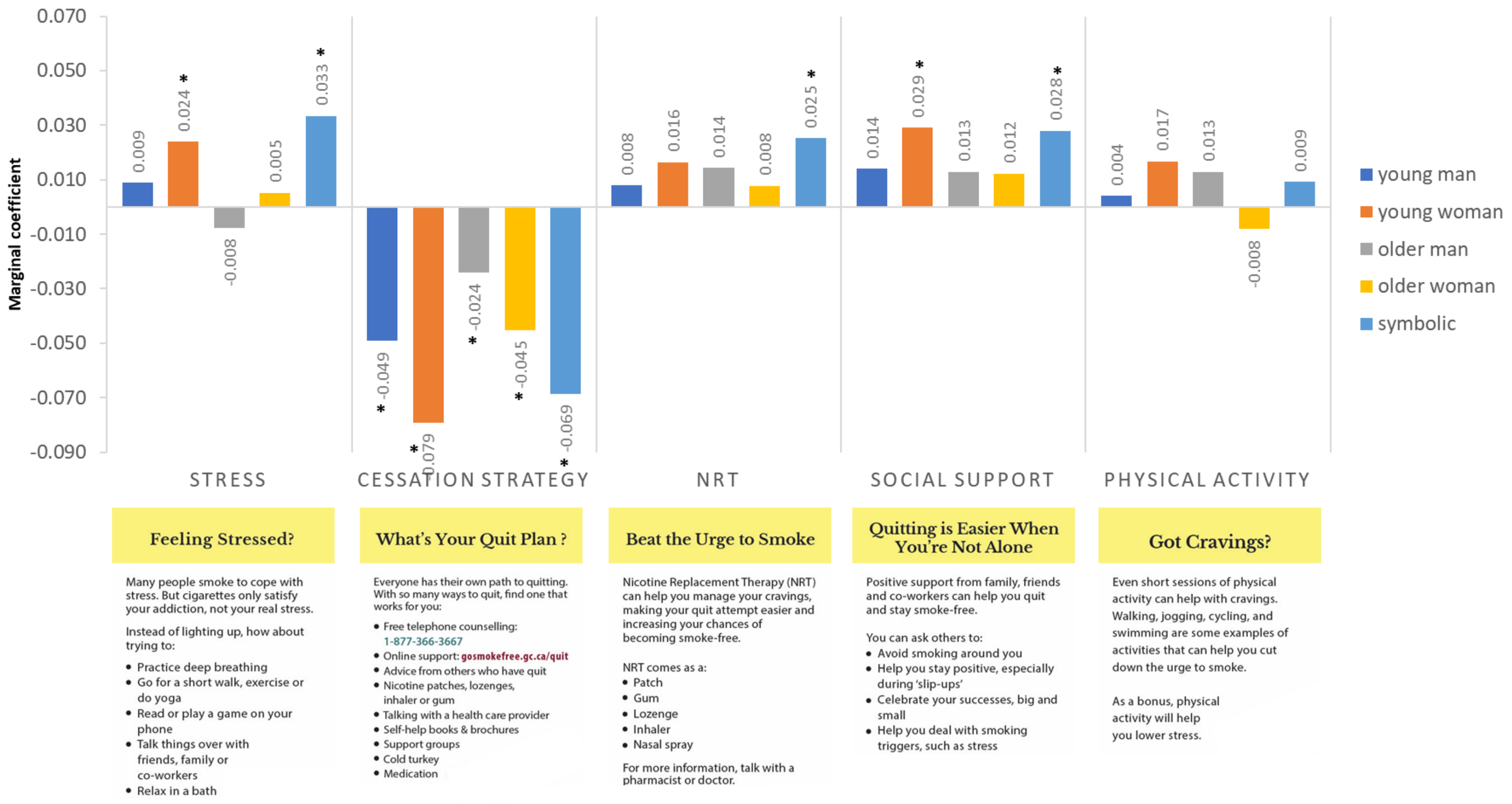

Figure S4. Interactions between message topic and image type; Results from DCE 2, general self-efficacy messages (* $p < 0.05$ for contrast with the grand mean).

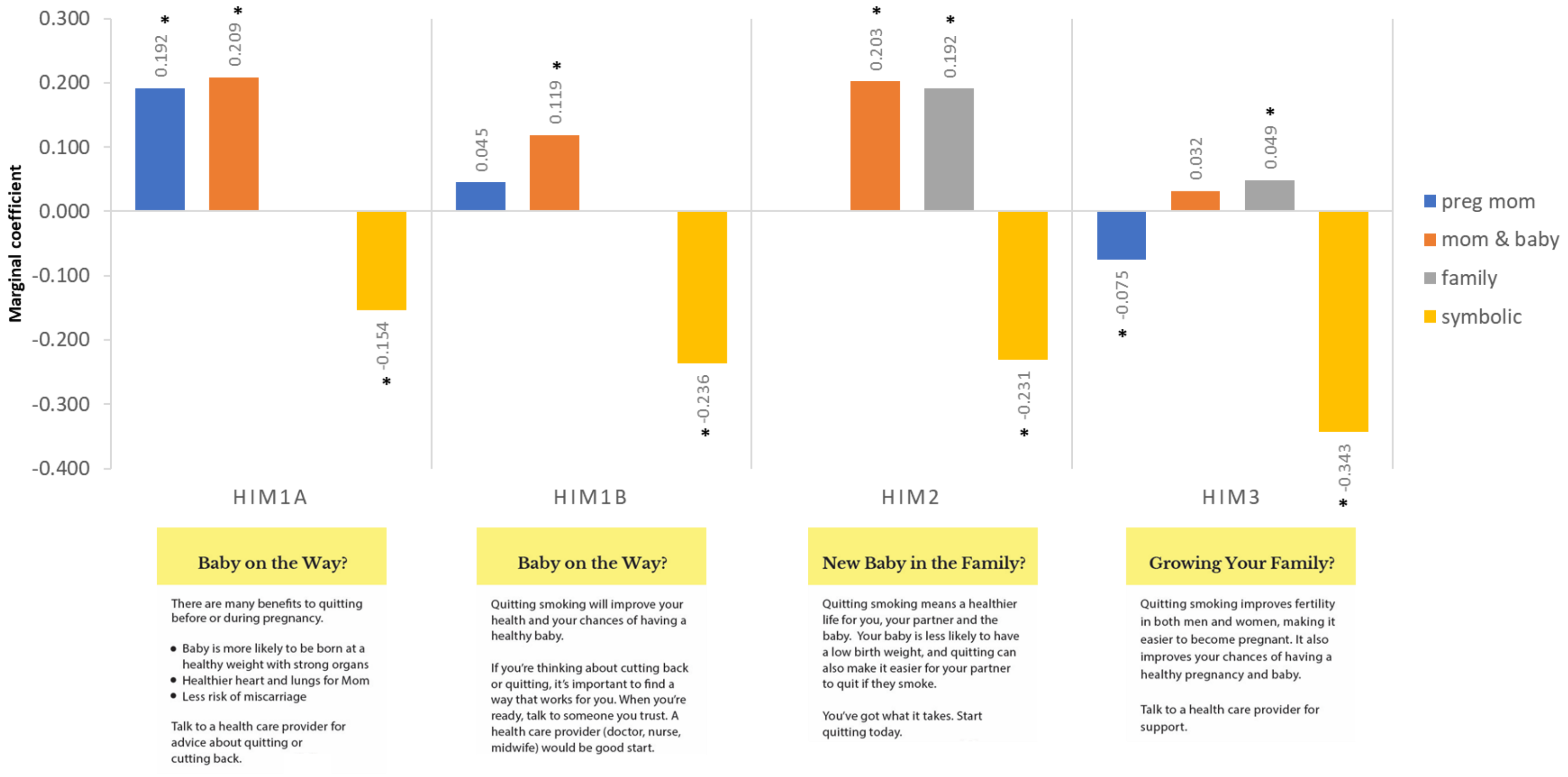

Figure S5. Interactions between message topic and image type; Results from DCE 3, reproductive health response efficacy messages (* $p < 0.05$ for contrast with the grand mean).
